# Systematic Review and Meta-analysis of Deaths Attributable to Antimicrobial Resistance, Latin America 

**DOI:** 10.3201/eid2911.230753

**Published:** 2023-11

**Authors:** Agustín Ciapponi, Ariel Bardach, María Macarena Sandoval, María Carolina Palermo, Emiliano Navarro, Carlos Espinal, Rodolfo Quirós

**Affiliations:** Centro de Investigaciones Epidemiológicas y Salud Pública (CIESP), Buenos Aires, Argentina (A. Ciapponi, A. Bardach);; Instituto de Efectividad Clínica y Sanitaria (IECS-CONICET), Buenos Aires (A. Ciapponi, A. Bardach, M.M. Sandoval, M.C. Palermo, E. Navarro);; Florida International University, Miami, Florida, USA (C. Espinal, R. Quirós);; Sanatorio Las Lomas, Buenos Aires (R. Quirós)

**Keywords:** antimicrobial resistance, bacteria, mortality, lethality, Latin America, Caribbean region, Argentina, Brazil, Colombia, methicillin-resistant Staphylococcus aureus

## Abstract

Antimicrobial resistance is a pressing global health concern, leading to 4.95 million deaths in 2019. We conducted a systematic review and meta-analysis to assess the lethality attributed to infections caused by multidrug-resistant organisms (MDROs) in Latin America and the Caribbean. A comprehensive search of major databases retrieved relevant studies from 2000–2022. We included 54 observational studies, primarily from Brazil, Argentina, and Colombia. The most commonly studied organism was methicillin-resistant *Staphylococcus aureus*. The overall unadjusted case fatality rate related to MDROs was 45.0%; higher adjusted lethality was observed in persons infected with MDROs than in those infected with other pathogens (adjusted odds ratio 1.93, 95% CI 1.58–2.37). A higher lethality rate was seen in patients who did not receive appropriate empirical treatment (odds ratio 2.27, 95% CI 1.44–3.56). These findings underscore the increased lethality associated with antimicrobial resistance in Latin America and the Caribbean.

Antimicrobial resistance (AMR) is a growing public health problem that affects health, the economy, and human development (*1*). A 2016 review of AMR showed that drug-resistant infections will kill 10 million persons annually by 2050 and cause a cumulative economic loss of US $100 trillion if proactive solutions to slow the rise of drug resistance are not implemented (*2*). Although some persons have criticized this forecast, numerous researchers agree that the spread of AMR is an urgent problem, one that will require a global, coordinated action plan to solve (*3*,*4*).

Recently, a study using statistical predictive models based on a comprehensive systematic review estimated 4.95 million deaths related to AMR, including 1.27 million deaths attributable to AMR, occurred across 204 countries and territories in 2019 (*5*). The highest burden of AMR is seen in low-resource settings. AMR was the third leading underlying cause of death for 2019 in the Institute for Health Metrics and Evaluation’s Global Burden of Disease study (https://www.healthdata.org/research-analysis/gbd). In addition, deaths attributable to AMR surpassed deaths caused by HIV, tuberculosis, and malaria. Understanding the effects of AMR is crucial for building policy resolutions, particularly regarding antimicrobial and diagnostic stewardship and infection prevention and control programs. This study, in which we defined attributable lethality as the excess lethality of patients with infections caused by resistant organisms compared with patients with infections caused by the same susceptible pathogens, represents an essential contribution to knowledge of the effects of AMR on lethality. However, because the estimations were performed for 2019, data related to the effects of the COVID-19 pandemic could not be part of this study. In addition, estimates of attributable lethality for the Latin America region were based principally on data from Brazil, Colombia, and Mexico; information from other countries in the region was limited (*5*). The incidence of multidrug-resistant organisms (MDROs) increased during the COVID-19 pandemic because of widespread use of antimicrobial drugs and breaches in infection control practices (*6*–*8*). During 2020–2021, Latin American and Caribbean (LAC) countries reported clinical emergence of carbapenemase-producing Enterobacterales that had not been previously characterized locally, increased prevalence of carbapenemases that had been previously detected, and coproduction of multiple carbapenemases in some isolates (*9*).

Several studies have estimated the effects of antibiotic resistance on incidence, deaths, length of hospital stay, and healthcare costs for MDROs (*1*,*2*,*10*), but systematic research on the effects of AMR in the LAC region for a wide range of bacteria and infections is lacking. We conducted a systematic review to address this evidence gap.

## Materials and Methods

### Search Strategy and Selection Criteria

We performed a systematic review and meta-analysis using Cochrane methods (*11*) and the PRISMA (*12*) statement for reporting systematic reviews and meta-analysis. We searched records published during January 1, 2000–March 29, 2022, in the databases CENTRAL (Cochrane Central Register of Controlled Trials), MEDLINE, Embase, LILACS (Latin American and Caribbean Health Sciences Literature), and CINAHL (Cumulative Index to Nursing and Allied Health Literature), without language restriction and with geographic scope of LAC countries (Appendix).

We included cohort studies, case–control studies, cross-sectional and control arms of randomized and quasi-randomized controlled trials, with >20 inpatient or outpatient participants, irrespective of age and sex, that assessed case-fatality rate (i.e., number of deaths among diagnosed cases only) within 30 days postinfection by any of the following resistant organisms: methicillin-resistant *Staphylococcus aureus* (MRSA), vancomycin-resistant *Enterococcus* spp. (VRE), extended-spectrum β-lactamase producing Enterobacterales (ESBL-E), carbapenem-resistant Enterobacterales (CRE, including *Klebsiella pneumoniae*, *Enterobacter*, *Escherichia coli*, *Proteus*, and *Serratia*), carbapenem-resistant *Pseudomonas aeruginosa* (CR-PA), carbapenem-resistant *Acinetobacter baumannii* (CR-AB), or azole/echinocandin-resistant *Candida* spp. (*1*). We have incorporated the MDRO category, which encompasses a diverse array of microorganisms (MRSA, VRE, ESBL-E, CRE, CR-PA, CR-AB). MDRO definitions and appropriate empirical treatment varied among primary studies. We accepted the definitions given by each study author.

We planned to include economic evaluations to assess resource use, including hospital stays and loss of health-related quality of life. We considered systematic reviews and meta-analyses only as sources for primary studies. When we found data or data subsets reported in >1 publication, we selected the most recent study or the study with the larger sample size. We searched databases containing proceedings of regional congresses and doctoral theses. We also consulted websites from the main regional medical societies, experts, and associations related to the topic (Appendix).

### Statistical Analysis

Pairs of reviewers independently selected articles by evaluating titles and abstracts of identified studies and then performing full-text review using Covidence software (https://www.covidence.org). One reviewer performed data extraction and a second verified data by using a prespecified extraction online form previously piloted in 10 studies. The same reviewers independently assessed the risk for bias using a checklist for observational studies developed by the US National Heart, Lung, and Blood Institute (https://www.nhlbi.nih.gov/health-topics/study-quality-assessment-tools). All authors resolved discrepancies by consensus.

We extracted study information consisting of type of publication, year of publication, authors, population, geographic location, study design, methods, pathogen–drug combinations, counterfactual data, and outcomes of interest. We classified population risk as high risk if they met >1 of the following characteristics: intensive care unit (ICU) setting or >50% of enrolled patients from ICU; median Charlson Comorbidity Index of >3 (if not reported, >50% of the patients with >1 comorbidity); or patients referred from high-risk wards (i.e., hematology, oncology, burns, transplantation, and infectious diseases, including HIV units). Otherwise, we classified the population as average risk. If study authors provided no data, we categorized the population risk as unknown.

To analyze our data, we used descriptive statistics and performed proportional meta-analysis using the random effects model whenever possible, employing methods to stabilize variance given the degree of expected heterogeneity. We applied the arcsine transformation to stabilize the variance of proportions using inverse arcsine variance weights for the random effects model with the metagen function (*13*,*14*). We used the restricted maximum-likelihood estimator to calculate the heterogeneity variance τ^2^ across the studies and I^2^ statistic as a measure of the proportion of the overall variation that was attributable to between-study heterogeneity (*15*,*16*).

The primary outcome was deaths attributable to AMR. Mortality rates describe the incidence of deaths among a specific population over a specific time. In this study, the population under investigation consisted of infected persons; although the accurate term is lethality (number of deaths among infected patients), we occasionally use the term mortality, which is more commonly used in the literature. We have included adjusted measures, such as odds ratio (OR), relative risk (RR), or hazard ratio (HR), when they were available for >10 events in the susceptible or resistance group. We included the longest time-point in-hospital lethality reported in each study in the analysis. We also performed a random-effects meta-analysis to estimate the pooled unadjusted OR when possible. Otherwise, we calculated ORs and 95% CIs using the information provided in each study. We report all effects estimates with a 95% CI. We describe the remaining results narratively and in tables.

We performed subgroup analysis by recruitment year and pathogen-drug combination. We performed a sensitivity analysis to assess the effect of risk for bias on the results of the primary analyses by limiting the analysis to a low risk for bias for the primary domains. We also used Knapp-Hartung adjustments to estimate 95% CIs around the pooled effect as a sensitivity analysis (*17*).

To further explore heterogeneity, we conducted a meta-regression analysis to examine whether adjusted and unadjusted effect estimates differed notably by year of study recruitment, population severity, resistance mechanisms or type of resistance, and appropriate empirical antibiotic treatment. We also planned a sensitivity analysis on the basis of the type of adjustment performed and considered it appropriate if the authors controlled in the final model >1 variable in each of these categories: variables related to the patients’ baseline status, variables related to the infection, and variables related to the treatment (*18*).

We visually inspected the funnel plot for asymmetry to assess publication bias and performed Begg’s test. We used R software version 4.0.3 for all analyses (*19*). The protocol of this study is registered in PROSPERO (https://www.crd.york.ac.uk/prospero; identification no. CRD42022322795).

## Results

**Figure 1:**
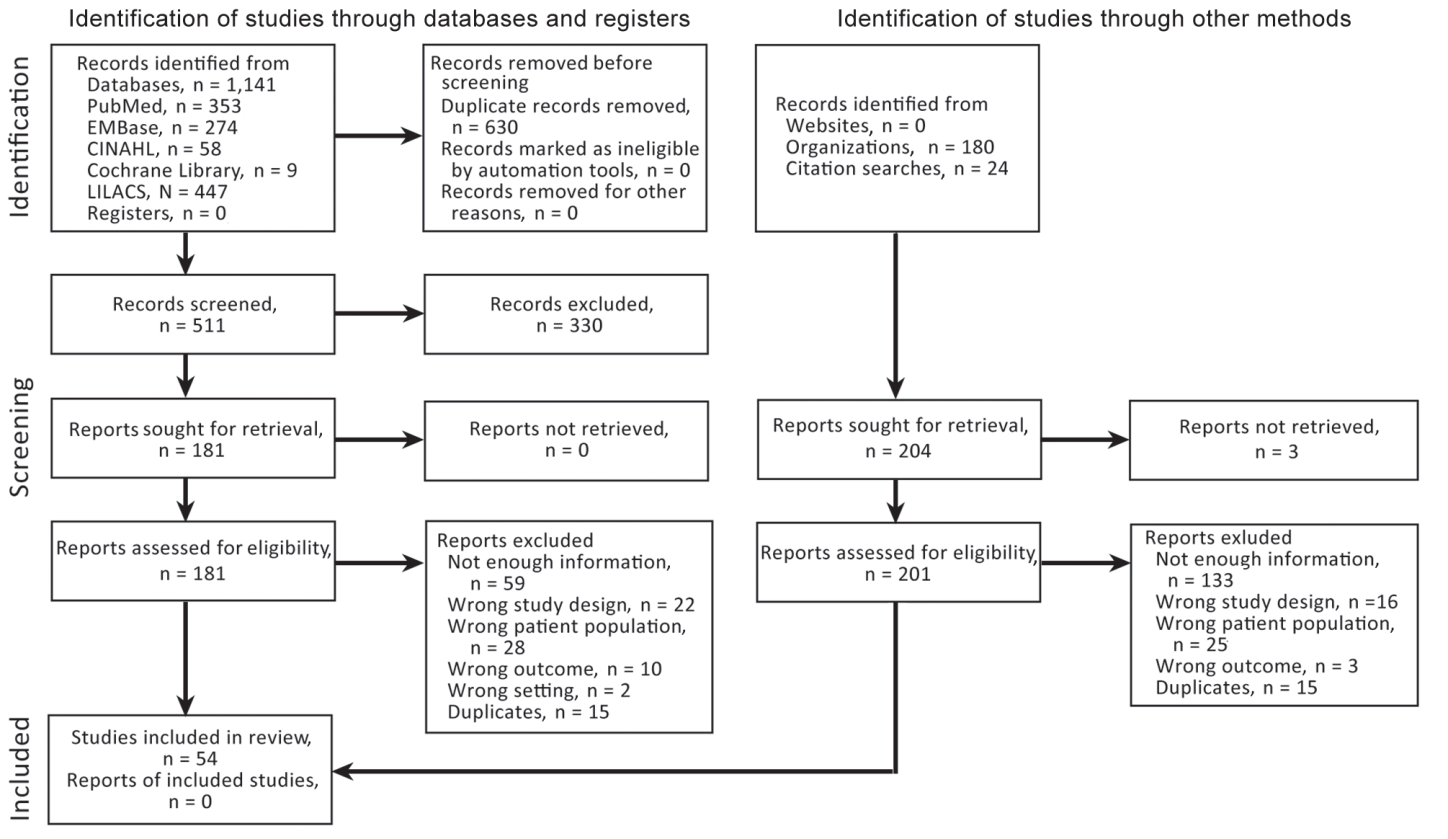
Flowchart demonstrating identification process of studies for systematic review and meta-analysis of deaths attributable to antimicrobial resistance, Latin America. CINAHL, Cumulative Index to Nursing and Allied Health Literature; LILACS, Latin American and Caribbean Health Sciences Literature).

We identified 1,141 records from databases and 204 records from other sources; after the selection process, 54 studies met our inclusion criteria (Figure 1). The articles were published during January 1, 2000–March 29, 2022; the inclusion period of participants was 1991–2020. We excluded 19 studies at the extraction stage (Appendix Table 6). Most included studies were cohort studies (50/54 [92.6%]); 36 were retrospective studies. The studies provided data on AMR mainly from Brazil (29 [53.7%]), Argentina (8 [14.8%]), Colombia (6 [11.1%]), and Mexico (6 [11.1%]). Of the 54 included studies, participants in 38 (70.4%) were adults (adults and elderly patients), and 6 (11.1%) studies included only children (neonates and pediatric patients). In the 49 studies that reported the source of patients, all participants were hospitalized, 18 (36.7%) consisted of ICU patients, and 20 (40.9%) included both ICU and non-ICU patients. High-risk populations were included in 43 (79.6%) studies. The most frequently evaluated individual microorganism was MRSA in 16 (29.6%) studies (Appendix Table 12). We identified a fair risk of bias in 24 (44.4%) of 54 studies (Appendix Tables 7, 8). We noted additional characteristics of individual included studies (Appendix Table 9).

**Figure 2:**
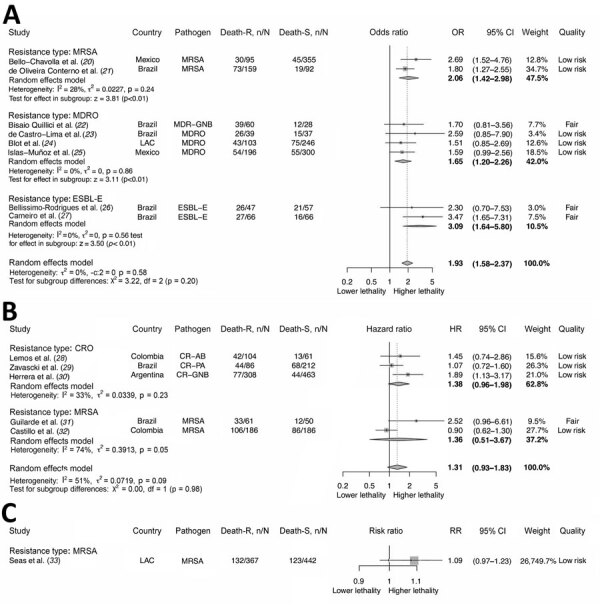
Association between antimicrobial resistance and lethality by the type of resistance in systematic review and meta-analysis of deaths attributable to antimicrobial resistance, Latin America. Adjusted measures are shown as adjusted odds ratio (A), adjusted hazard ratio (B), and adjusted risk ratio (C). Death-R indicates death in the resistant group; Death-S indicates death in the susceptible group. Error bars indicate 95% CIs. CR-AB, carbapenem-resistant *Acinetobacter baumannii*; CRE, carbapenem-resistant Enterobacterales; CR-GNB, carbapenem-resistant gram-negative bacteria (including CRE, CR-PA, CR-AB); CR-PA, carbapenem-resistant *Pseudomonas aeruginosa*; CRO, carbapenem-resistant organisms; ESBL-E, extended-spectrum β-lactamase–producing Enterobacterales; HR, hazard ratio; LAC, Latin American and Caribbean; MDR-GNB, multidrug-resistant gram-negative bacilli (including ESBL-E, CRE, CR-PA, CR-AB); MDRO, multidrug-resistant organisms (including MRSA, vancomycin-resistant *Enterococcus*, ESLB-E, CRE, CR-PA, CR-AB); MRSA, methicillin-resistant *Staphylococcus aureus*; OR, odds ratio; RR, risk ratio.

We assessed lethality and association measures of the individual studies (Appendix Table 13). The overall unadjusted case-fatality rate related to MDRO was 45.0% (95% CI 40.0–50.0; I^2^ 85.0%) (Appendix Figure 1). We found higher lethality among participants infected with MDRO than among participants infected with nonresistant organisms, grouped according to the type of resistance (pooled adjusted OR [aOR] 1.93, 95% CI 1.58–2.37; I^2^ 0%) (Figure 2). Although that trend was maintained in studies that reported RR or HR as adjusted measures, the difference was not statistically significant. We found no evidence of publication bias among studies reporting aOR or adjusted HR (Appendix Figures 2, 3).

**Figure 3:**
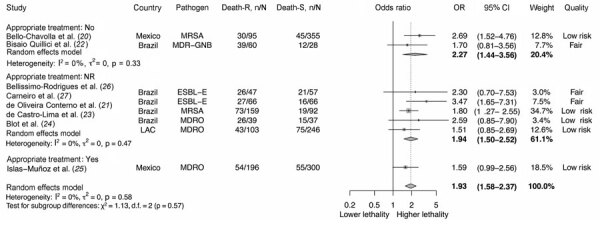
Adjusted odds ratios between antimicrobial resistance and lethality by appropriate empirical antibiotic treatment (considering the definition of appropriate empirical treatment given by each author) in systematic review and meta-analysis of deaths attributable to antimicrobial resistance, Latin America. Death-R indicates death in the resistant group; Death-S indicates death in the susceptible group. Error bars indicate 95% CIs. CR-AB, carbapenem-resistant *Acinetobacter baumannii*; CRE, carbapenem-resistant Enterobacterales; CR-PA, carbapenem-resistant *Pseudomonas aeruginosa*; ESBL-E, extended-spectrum β-lactamase–producing Enterobacterales; MDR-GNB, multidrug-resistant gram-negative bacilli (including ESBL-E, CRE, CR-PA, CR-AB); MDRO, multidrug-resistant organisms (including MRSA, vancomycin-resistant Enterococcus, ESLB-E, CRE, CR-PA, CR-AB); MRSA, methicillin-resistant *Staphylococcus aureus*; NR, not reported; OR, odds ratio.

Higher lethality was also observed in those who did not receive appropriate empirical treatment (OR 2.27, 95% CI 1.44–3.56) than in those who did (OR 1.59, 95% CI 0.99–2.56), although the test for subgroup differences was not statistically significant (p = 0.57). We also found no statistically significant difference (p = 0.75) between resistance and lethality in those studies that included appropriate empiric antibiotic treatment as a covariate in the adjusted model (Figure 3; Appendix Figure 4).

**Figure 4:**
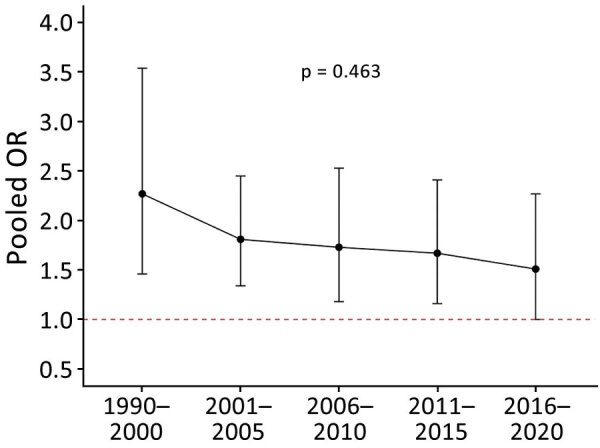
Pooled unadjusted OR between antimicrobial resistance and lethality by calendar recruitment period in systematic review and meta-analysis of deaths attributable to antimicrobial resistance, Latin America. Data points depict pooled lethality estimates from random effects meta-analysis models. Error bars indicate 95% CIs. OR, odds ratio.

We report the association between unadjusted lethality and type of resistance (Table). The pooled unadjusted lethality associated with resistant infections was significantly higher (OR 1.86, 95% CI 1.55–2.23) than that associated with susceptible infections but with high heterogeneity (I^2^ 71%) (Appendix Figure 5). We identified a downward trend (p = 0.463) of this pooled OR of lethality over time (Figure 4). We also presented a forest plot of unadjusted OR for lethality by year of study recruitment (Appendix Figure 6). The results of a meta-regression analysis showed no significant differences in effect estimates (Appendix Table 10).

**Table:** Pooled unadjusted odds ratio for the association between antimicrobial resistance and lethality by type of resistance, Latin America*

Type of resistance	OR (95% CI)	I^2^
Carbapenem-resistance	2.86 (2.07–3.95)	61%
Extended-spectrum β-lactamase	1.28 (0.95–1.74)	38%
Methicillin-resistance	1.78 (1.29–2.45)	63%
MDRO†	1.64 (1.16–2.30)	68%
Azol-resistant	1.41 (0.59–3.35)	-
Vancomycin-resistant	4.09 (2.40–6.97)	0%
Random effect model	1.86 (1.55–2.23)	71%

*MDRO, multidrug-resistant organisms; OR, pooled odds ratio. †Multidrug-resistant organisms include methicillin-resistant *Staphylococcus aureus*, Vancomycin-resistant *Enterococcus* spp, extended-spectrum β-lactamase producing Enterobacterales, carbapenem-resistant Enterobacterales (including *Klebsiella, Enterobacter, Escherichia coli, Proteus, Serratia*), carbapenem-resistant *Pseudomonas aeruginosa*, carbapenem-resistant *Acinetobacter baumannii* and azole/echinocandin-resistant *Candida* spp.

We analyzed the difference between the pooled unadjusted and adjusted lethality associated with resistance between studies that reported both measures (Appendix Figure 7). The magnitude of the effect was larger when looking at the unadjusted measures, but the differences between subgroups were not statistically significant (p = 0.360).

We performed a sensitivity analysis based on the type of multivariate model adjustment reported in the studies. Although the aOR was larger when the adjustment was appropriate, we found no statistically significant subgroup differences (p = 0.56) (Appendix Figure 8). As a sensitivity analysis, we report the 95% CI around the pooled effect with Knapp-Hartung adjustments in those studies that report adjust measures. We found no major differences with or without this method (Appendix Table 11).

In all but 2 reports where hospitalization stay was documented, patients with resistant microorganisms exhibited longer length of stay relative to those with susceptible strains (Appendix Table 9). We did not find any information related to loss of health-related quality of life attributable to MDRO in the region. Length of stay was not reported because data were scarce and heterogeneous.

## Discussion

This systematic review and meta-analysis offers a thorough and current evaluation of how infection with a wide range of antimicrobial-resistant bacteria affects the lethality rates for infectious diseases during hospitalization in LAC countries. We established the unadjusted and adjusted lethality attributable to MDRO within this region. A previous study reported estimations using predictive statistical modeling to produce estimates of AMR burden for all locations, including for locations with no data. However, the methodologic approach used in this study differed substantially (*5*).

Although unadjusted case-fatality rates varied across different MDROs, the lowest values were observed for ESLB-E. That finding might be because of the increased use of carbapenems as appropriate initial empirical treatments, especially for healthcare-associated infections, which some studies have demonstrated in the region (*34*).

Similar to previous researchers (*35*,*36*), we also report that drug resistance might lead to an increased attributable risk for death. However, our findings should be interpreted with caution because of substantial heterogeneity in effect estimates across studies and other methodologic limitations. The heterogeneity was partially explained by the fact that some studies were adjusted for confounding variables, but others were not. When we analyzed studies adjusted for confounding variables separately, the results for each group were no longer heterogeneous. The adjustment decreased the association strength, although the subgroup differences were not statistically significant. The downward trend of pooled unadjusted lethality OR and resistance by calendar recruitment period was not statistically significant, but this finding still might reflect a better understanding of the resistance mechanisms and an improved empirical treatment.

As in previous reports (*37*,*38*), our report found 2 times higher attributable lethality associated with MRSA infections than with non-MRSA infections. As in our study, the heterogeneity was explained by the fact that some studies were adjusted for confounding variables, but others were not. When those studies that were adjusted for confounding variables were analyzed separately from studies that were not adjusted, the results for each group were no longer heterogeneous (Appendix Figure 7). Of note, several studies have identified the association of inappropriate empirical antibiotic treatment with increased lethality among patients with MRSA bacteremia (*39*,*40*).

In our study, patients with VRE infections were 4-fold (unadjusted) more likely to die than patients infected with vancomycin-susceptible *Enterococcus* spp. A previous meta-analysis indicated that vancomycin resistance was an independent predictor of death among patients with enterococcal bacteremia (2.5-fold adjusted) (*41*). Some plausible explanations for this association difference, besides the adjustment of the last estimation, might include type of infection, suboptimal activity, or dosing among the antimicrobials used against VRE, a systematic delay in the initiation of antimicrobial agents active against VRE, and differences in intrinsic virulence among vancomycin-resistant and vancomycin-susceptible species of enterococci.

In our study, infections by ESBL-E were associated with higher lethality than for non–ESBL-E. Other studies have found that ESBL-E bacteremia is associated with higher lethality than bacteremia with non–ESBL-E, although the estimate of this association is affected by adjustment procedures. Adjustment for adequate empirical therapy or delay in effective therapy leads to reduced ORs, indicating that higher lethality is likely to be partly mediated through this phenomenon (*42*–*44*).

We found a significant attributable lethality associated with carbapenem-resistant organisms in 11 studies included in our meta-analysis. A previous study showed that KPC-producing *K. pneumoniae* was independently associated with 3 times higher in-hospital lethality (*45*).

A meta-analysis of 15 studies consisting of 3,201 cases of *P. aeruginosa* infection demonstrated a 2-fold higher lethality rate among patients infected with the multidrug-resistant strain than those with a non–multidrug-resistant strain, especially in patients with bloodstream infection, immunosuppression, and inadequate antimicrobial therapy (*35*). Other meta-analyses showed that appropriate initial antibiotic therapy was associated with lower unadjusted lethality for *P. aeruginosa* infections than was inappropriate initial antibiotic therapy. The association with lethality persisted in sensitivity meta-analysis of low-risk bias studies (*46*).

In a meta-analysis that included 16 observational studies, patients with CR-AB had a significantly 2-fold higher risk for lethality than patients with non–carbapenem-resistant strain in the pooled analysis, although substantial heterogeneity was evident. The association remained significant in the pooled aOR of 10 studies. Compared with patients with non–carbapenem-resistant strains, patients with CR-AB were more likely to have a severe underlying illness and to receive inappropriate empirical antimicrobial treatment, which increases the risk for lethality (*47*).

In our meta-analysis, 4 studies evaluating attributable lethality showed that MDRO had significantly higher lethality than non-MDRO. Although different microorganisms and site infections were represented, we did not find statistical heterogeneity.

For gram-negative infections, a meta-analysis showed that lethality was higher in patients with multidrug-resistant infections than those with non–multidrug-resistant infections (*48*). The meta-analysis demonstrated that septic shock, ICU stay, pneumonia, isolation of multidrug-resistant gram-negative bacteria, inappropriate empirical and definitive treatment, and male sex were more common in patients who died than patients who survived (*48*). In addition, several studies have reported inappropriate empirical and definitive treatment as independent variables associated with attributable lethality (*35*,*39*–*44*,*46*–*49*)

As the incidence of AMR rises, a corresponding increase in the likelihood of inappropriate empirical treatment occurs. Our meta-analysis revealed that persons who did not receive appropriate empirical treatment had a higher lethality rate than those who did. However, the lack of information regarding the adequacy of antimicrobial therapy in many studies might explain the absence of statistically significant differences between subgroups.

The first limitation of our study is that of the 41 cohort studies included, only 14 were prospective. Incomplete collection in surveys in retrospective cohort studies limits confidence in their estimates. Other limitations include the lack of data for most countries in the LAC region and the number of included studies with small samples. For example, most data were obtained from a few tertiary centers in each country, which are likely to report higher rates of resistance than their national averages. Methods for reporting, collection, and analysis might also differ among laboratories, countries, and surveillance networks. Other limitations included the type of infections (which might vary across included studies), the type of antimicrobial drugs administered for the infection, the type of bacteria, and the mechanism of resistance, which might lead to differences in lethality. 

In conclusion, our systematic review and meta-analysis demonstrate that MDROs are associated with higher attributable lethality across different periods in LAC than sensitive organisms, even after adjusting for confounding variables. More studies on AMR-attributable lethality would be needed in the region, with adjustment by confounders and larger sample sizes. Rather than relying solely on new drug development to address the problem of AMR, we should focus efforts on preventing the emergence and transmission of these organisms through the One Health initiative, principally in low-income settings (*50*). Future studies that involve many healthcare centers and that adjust for potential confounding variables should be undertaken to address the impact of AMR. In addition, expanding microbiology laboratory capacity and data collection systems are necessary to improve our understanding of this critical human health threat. 

Appendix Additional information about deaths attributable to antimicrobial resistance, Latin America.
