# Supplementary material for: Systematic Review and Meta-analysis of Deaths Attributable to Antimicrobial Resistance, Latin America
Source: Emerg Infect Dis. 2023 Nov;29(11):2335–44. doi: 10.3201/eid2911.230753 (PMC10617342; doi:10.3201/eid2911.230753)
Supplement: Appendix — Additional information about deaths attributable to antimicrobial resistance, Latin America. [file 23-0753-Techapp-s1.pdf]

Article DOI: https://doi.org/10.3201/eid2911.230753

EID cannot ensure accessibility for supplementary materials supplied by authors. Readers who have difficulty accessing supplementary content should contact the authors for assistance.

Systematic Review and Meta-analysis of Deaths Attributable to Antimicrobial Resistance, Latin America

Appendix

Search Strategy

Appendix Table 1. Terms used in PubMed search, 26 March 2022

Search	Query
#67	#4 AND #29 AND #50 AND #65 AND #66 Filters: from 2000/1/1 - 3000/12/12
#66	(Americas[MeSH Terms:noexp] OR Latin America[Mesh] OR Latin America*[tiab] OR Latinamerica*[tiab] OR Latinoamerica*[tiab] OR Hispanoamerica*[tiab] OR Iberoamerica*[tiab] OR Ibero Americ*[tiab] OR Panamerican*[tiab] OR Central America[Mesh] OR Central America*[tiab] OR Centroamerica*[tiab] OR Mesoamerica*[tiab] OR Meso America*[tiab] OR Middle America*[tiab] OR South America[Mesh] OR South America*[tiab] OR Southamerica*[tiab] OR Sudamerica*[tiab] OR “America del sur”[tiab] OR Caribbean Region[Mesh] OR Caribbean[tiab] OR Caribe*[tiab] OR West Indies[Mesh] OR West Indi*[tiab] OR Antill*[tiab] OR Indians, South American[Mesh] OR Indians, Central American[Mesh] OR Amerindian*[tiab] OR Indians[tiab] OR American Indian*[tiab] OR Native America*[tiab] OR Patagoni*[tiab] OR Andes[tiab] OR Andean*[tiab] OR Amazon*[tiab] OR Argentin*[ad] OR Argentin*[tiab] OR Argentina[pl] OR Bolivia*[ad] OR Bolivia*[tiab] OR Bolivia[pl] OR Brazil*[ad] OR Brasil*[ad] OR Brazil*[tiab] OR Brasil*[tiab] OR Brazil[pl] OR Colombia*[ad] OR Colombia*[tiab] OR Colombia[pl] OR Chile*[ad] OR Chile*[tiab] OR Chile[pl] OR Ecuador*[ad] OR Ecuator*[ad] OR Ecuador*[tiab] OR Ecuador[pl] OR Guiana*[ad] OR Guiana*[tiab] OR French Guiana[pl] OR Guyan*[ad] OR Guyan*[tiab] OR Guyana[pl] OR Paraguay*[ad] OR Paraguay*[tiab] OR Paraguay[pl] OR Peru*[ad] OR Peru*[tiab] OR Peru[pl] OR Surinam*[ad] OR Surinam*[tiab] OR Surinam*[pl] OR Uruguay*[ad] OR Uruguay*[tiab] OR Uruguay[pl] OR Venez*[ad] OR Venez*[tiab] OR Venezuela[pl] OR Belize*[ad] OR Belize*[tiab] OR Belize[pl] OR Costa Ric*[ad] OR Costarric*[ad] OR Costaric*[ad] OR Costa Ric*[tiab] OR Costarric*[tiab] OR Costaric*[tiab] OR Costa Rica[pl] OR Salvador*[ad] OR Salvador*[tiab] OR El Salvador[pl] OR Guatemal*[ad] OR Guatemal*[tiab] OR Guatemala[pl] OR Hondur*[ad] OR Hondur*[tiab] OR Honduras[pl] OR Nicaragu*[ad] OR Nicaragu*[tiab] OR Nicaragua[pl] OR Panam*[ad] OR Panam*[tiab] OR Panama[pl] OR Mexico[Mesh] OR Mexic*[ad] OR Mexic*[tiab] OR Mejic*[tiab] OR Mexico[pl] OR Baham*[ad] OR Baham*[tiab] OR Bahamas[pl] OR Cuba*[ad] OR Cuba*[tiab] OR Cuba[pl] OR Dominic*[ad] OR Dominic*[tiab] OR Dominican Republic[pl] OR Haiti*[ad] OR Haiti*[tiab] OR Haiti[pl] OR Jamaic*[ad] OR Jamaic*[tiab] OR Jamaica[pl] OR Puerto Rico[Mesh] OR Puerto Ric*[tiab] OR Puertorric*[tiab] OR Puertoric*[tiab])
#65	#51 OR #52 OR #53 OR #54 OR #55 OR #56 OR #57 OR #58 OR #59 OR #60 OR #61 OR #62 OR #63 OR #64
#64	Hospital Stay*[tiab]
#63	“Length of Stay”[tiab]
#62	Stay Length*[tiab]
#61	Length of Stay[Mesh]
#60	DALY*[tiab]
#59	Disability Adjusted[tiab]
#58	Adjusted Life[tiab]
#57	QALY*[tiab]
#56	Quality Adjusted[tiab]
#55	Quality-Adjusted Life Years[Mesh]
#54	Fatality Rate*[tiab]
#53	Death Rate*[tiab]
#52	Mortalit*[tiab]

Search	Query
#51	Mortality[Mesh]
#50	#30 OR #31 OR #32 OR #33 OR #34 OR #35 OR #36 OR #37 OR #38 OR #39 OR #40 OR #41 OR #42 OR #43 OR #44 OR #45 OR #46 OR #47 OR #48 OR #49
#49	ICU Infect*[tiab]
#48	ICU Associated[tiab]
#47	ICU Acquired[tiab]
#46	Community Infect*[tiab]
#45	Community Associated[tiab]
#44	Community Acquired[tiab]
#43	Healthcare Associated[tiab]
#42	Healthcare Infect*[tiab]
#41	Healthcare Acquired[tiab]
#40	Intrahospital Acquired[tiab]
#39	Intrahospital Associated[tiab]
#38	Intrahospital Infect*[tiab]
#37	Hospital Infect*[tiab]
#36	Hospital Associated[tiab]
#35	Hospital Acquired[tiab]
#34	Nosocomial Acquired[tiab]
#33	Nosocomial Associated[tiab]
#32	Nosocomial Infect*[tiab]
#31	Cross infect*[tiab]
#30	Cross Infection[Mesh]
#29	#5 OR #6 OR #7 OR #8 OR #9 OR #10 OR #11 OR #12 OR #13 OR #14 OR #15 OR #16 OR #17 OR #18 OR #19 OR #20 OR #21 OR #22 OR #23 OR #24 OR #25 OR #26 OR #27 OR #28
#28	Enterobacter*[tiab]
#27	Enterobacteriaceae[Mesh]
#26	Enterococc*[tiab]
#25	Enterococcus[Mesh]
#24	Staphylococcus Aure*[tiab]
#23	Staphylococcus Aureus[Mesh]
#22	Herellea*[tiab]
#21	Acinetobacter*[tiab]
#20	Acinetobacter[Mesh]
#19	Pseudomona*[tiab]
#18	Pseudomonas[Mesh]
#17	Jadinii[tiab]
#16	Monilia*[tiab]
#15	Candid*[tiab]
#14	Candida[Mesh]
#13	Carbapenemase-Producing Enterobacter*[tiab]
#12	Carbapenem Resist*[tiab]
#11	Carbapenem-Resistant Enterobacteriaceae[Mesh]
#10	Cephalosporin Resist*[tiab]
#9	Vancomycin Resist*[tiab]
#8	Vancomycin-Resistant Enterococci[Mesh]
#7	MRSA[tiab]
#6	Methicillin Resist*[tiab]
#5	Methicillin-Resistant Staphylococcus Aureus[Mesh]
#4	#1 OR #2 OR #3
#3	AMR[tiab]
#2	Resist*[all]
#1	Drug Resistance, Microbial[Mesh]

Appendix Table 2. Terms used in EMBase (OVID) search, 28 March 2022 (Embase Classic+Embase <1947 to 2022 March 28>)

#	Query
1	exp antibiotic resistance/
2	Resist*.mp.
3	AMR.ti,ab.
4	or/1–3
5	exp methicillin resistant Staphylococcus aureus/
6	(Methicillin adj3 Resist*).ti,ab.
7	MRSA.ti,ab.
8	exp vancomycin resistant Enterococcus/
9	(Vancomycin adj3 Resist*).ti,ab.
10	(Cephalosporin adj3 Resist*).ti,ab.
11	exp carbapenem-resistant Enterobacteriaceae/
12	(Carbapenem adj3 Resist*).ti,ab.
13	(Carbapenemase-Producing adj3 Enterobacter*).ti,ab.
14	exp Candida/
15	Candid*.ti,ab.
16	Monilia*.ti,ab.
17	Jadinii.ti,ab.
18	exp Pseudomonas/
19	Pseudomona*.ti,ab.
20	exp Acinetobacter/
21	Acinetobacter*.ti,ab.
22	Herellea*.ti,ab.
23	exp Staphylococcus aureus/
24	Staphylococcus Aure*.ti,ab.
25	exp Enterococcus/
26	Enterococc*.ti,ab.
27	exp Enterobacteriaceae/
28	Enterobacter*.ti,ab.
29	or/5–28
30	exp cross infection/
31	(Cross* adj1 infect*).ti,ab.
32	(Nosocomial adj1 Infect*).ti,ab.
33	(Nosocomial adj1 Associated).ti,ab.
34	(Nosocomial adj1 Acquired).ti,ab.
35	(Hospital* adj1 Acquired).ti,ab.
36	(Hospital* adj1 Associated).ti,ab.
37	(Hospital* adj1 Infect*).ti,ab.
38	(Intra?hospital adj1 Infect*).ti,ab.
39	(Intra?hospital adj1 Associated).ti,ab.
40	(Intra?hospital adj1 Acquired).ti,ab.
41	(Health?care adj1 Acquired).ti,ab.
42	(Health?care adj1 Infect*).ti,ab.
43	(Health?care adj1 Associated).ti,ab.
44	(Community adj1 Acquired).ti,ab.
45	(Community adj1 Associated).ti,ab.
46	(Community adj1 Infect*).ti,ab.
47	(ICU adj1 Acquired).ti,ab.
48	(ICU adj1 Associated).ti,ab.
49	(ICU adj1 Infect*).ti,ab.
50	or/30–49
51	exp mortality/
52	Mortalit*.ti,ab.
53	Death Rate*.ti,ab.
54	Fatality Rate*.ti,ab.
55	exp quality adjusted life year/
56	(Quality adj1 Adjusted).ti,ab.
57	QALY*.ti,ab.
58	exp disability-adjusted life year/
59	(Disability adj1 Adjusted).ti,ab.
60	(Adjusted adj1 Life).ti,ab.
61	DALY*.ti,ab.
62	exp “length of stay”/
63	(Stay adj3 Length*).ti,ab.
64	(Hospital* adj3 Stay*).ti,ab.
65	or/51–64
66	(exp South/ and Central America/) or (Latin adj1 America*).ti,ab. or Latinamerica*.ti,ab. or Latinoamerica*.ti,ab. or Hispanoamerica.ti,ab. or Iberoamerica*.ti,ab. or (Ibero adj1 Americ*).ti,ab. or Panamerica*.ti,ab. or (South adj1 America*).ti,ab. or Southamerica*.ti,ab. or Sudamerica*.ti,ab. or (America adj1 Sur).ti,ab. or (Central adj1

#	Query
	America*).ti,ab. or Centroamerica*.ti,ab. or Mesoamerica*.ti,ab. or (Meso adj1 America*).ti,ab. or (Middle adj1 America*).ti,ab. or exp Caribbean Islands/ or Caribbean*.ti,ab. or Caribe*.ti,ab. or (West adj1 Indi*).ti,ab. or Antill*.ti,ab. or exp American indian/ or Amerindian*.ti,ab. or Indians.ti,ab. or (Native adj1 America*).ti,ab. or Patagoni*.ti,ab. or Andes.ti,ab. or Andean*.ti,ab. or Amazon*.ti,ab. or exp Argentina/ or Argentin*.ti,ab. or exp Bolivia/ or Bolivia*.ti,ab. or exp Brazil/ or Brazil*.ti,ab. or Brasil*.ti,ab. or exp Colombia/ or Colombia*.ti,ab. or exp Chile/ or Chile*.ti,ab. or exp Ecuador/ or Ecuador*.ti,ab. or exp French Guiana/ or Guiana*.ti,ab. or exp Guyana/ or Guyan*.ti,ab. or exp Paraguay/ or Paraguay*.ti,ab. or exp Peru/ or Peru*.ti,ab. or exp Suriname/ or Surinam*.ti,ab. or exp Uruguay/ or Uruguay*.ti,ab. or exp Venezuela/ or Venez*.ti,ab. or exp Belize/ or Beliz*.ti,ab. or exp Costa Rica/ or (Costa adj1 Rica).ti,ab. or Costarric*.ti,ab. or Costaric*.ti,ab. or exp El salvador/ or Salvador*.ti,ab. or exp Guatemala/ or Guatemal*.ti,ab. or exp Honduras/ or Hondur*.ti,ab. or exp Nicaragua/ or Nicaragu*.ti,ab. or exp Panama/ or Panam*.ti,ab. or exp Mexico/ or Mexic*.ti,ab. or exp Cuba/ or Cuba*.ti,ab. or exp Dominican Republic/ or Dominica*.ti,ab. or exp Haiti/ or Haiti*.ti,ab. or exp Jamaic/ or Jamaic*.ti,ab. or exp Puerto Rico/ or (Puerto adj1 Ric*).ti,ab. or Puertoric*.ti,ab. or Puertorric*.ti,ab.
67	4 and 29 and 50 and 65 and 66
68	limit 67 to yr = "2000 -Current"

Appendix Table 3. Terms used in CINAHL Complete (EBSCO) search, 29 March 2022

#	Query
S70	S4 AND S31 AND S51 AND S66 AND S69 Limiters - Published Date: 20000101–20220331
S69	S67 OR S68
S68	AB (Latin N1 America*) OR Latinamerica* OR Latinoamerica* OR Latin* OR Hispanic Americans OR Iberoamerica* OR (Ibero N1 Americ*) OR Panamerican* OR (Central N1 America*) OR Centroamerica* OR Mesoamerica* OR (Meso N1 America*) OR (Middle N1 America*) OR (South N1 America*) OR Southamerica* OR Sudamerica* OR (America N1 Sur) OR Caribbean OR Caribe* OR (West N1 Indi*) OR Antill* OR Amerindian* OR Indians OR (American N1 Indian*) OR (Native N1 America*) OR Patagoni* OR Andes OR Andean* OR Amazon* OR Argentin* OR Bolivia* OR Brazil* OR Brasil* Colombia* OR Colombia* OR Colombia OR Chile* OR Ecuador* OR Guiana* OR Guyan* OR Guyan* OR Paraguay* OR Paraguay* OR Peru* OR Surinam* OR Surinam* OR Uruguay* OR Venez* OR Belize* OR (Costa N1 Ric*) OR Costarric* OR Costaric* OR Costa Ric* OR Costarric* OR Salvador* OR Salvador* OR Guatemal* OR Guatemal* OR Guatemala OR Hondur* OR Nicaragu* OR Panam* OR Mexic* OR Cuba* OR Dominic* OR Dominic* OR Haiti* OR Jamaic* OR (Puerto N1 Ric*) OR Puertorric* OR Puertoric*
S67	TI (Latin N1 America*) OR Latinamerica* OR Latinoamerica* OR Latin* OR Hispanic Americans OR Iberoamerica* OR (Ibero N1 Americ*) OR Panamerican* OR (Central N1 America*) OR Centroamerica* OR Mesoamerica* OR (Meso N1 America*) OR (Middle N1 America*) OR (South N1 America*) OR Southamerica* OR Sudamerica* OR (America N1 Sur) OR Caribbean OR Caribe* OR (West N1 Indi*) OR Antill* OR Amerindian* OR Indians OR (American N1 Indian*) OR (Native N1 America*) OR Patagoni* OR Andes OR Andean* OR Amazon* OR Argentin* OR Bolivia* OR Brazil* OR Brasil* Colombia* OR Colombia* OR Colombia OR Chile* OR Ecuador* OR Guiana* OR Guyan* OR Guyan* OR Paraguay* OR Paraguay* OR Peru* OR Surinam* OR Surinam* OR Uruguay* OR Venez* OR Belize* OR (Costa N1 Ric*) OR Costarric* OR Costaric* OR Costa Ric* OR Costarric* OR Salvador* OR Salvador* OR Guatemal* OR Guatemal* OR Guatemala OR Hondur* OR Nicaragu* OR Panam* OR Mexic* OR Cuba* OR Dominic* OR Dominic* OR Haiti* OR Jamaic* OR (Puerto N1 Ric*) OR Puertorric* OR Puertoric*
S66	S52 OR S53 OR S54 OR S55 OR S56 OR S57 OR S58 OR S59 OR S60 OR S61 OR S62 OR S63 OR S64 OR S65
S65	TI (Stay N3 Hospital*) OR AB (Stay N3 Hospital*)
S64	TI (Stay N3 Length*) OR AB (Stay N3 Length*)
S63	(MH "Length of Stay")
S62	TI DALY* OR AB DALY*
S61	TI (Disability N1 Adjusted) OR AB (Disability N1 Adjusted)
S60	(MM "Disability-Adjusted Life Years")
S59	TI (Adjusted N1 Life) OR AB (Adjusted N1 Life)
S58	TI QALY* OR AB QALY*
S57	TI (Quality N1 Adjusted) OR AB (Quality N1 Adjusted)
S56	(MM "Quality-Adjusted Life Years")
S55	TI (Fatality N1 Rate*) OR AB (Fatality N1 Rate*)
S54	TI (Death N1 Rate*) OR AB (Death N1 Rate*)
S53	TI Mortalit* OR AB Mortalit*
S52	(MH "Mortality+")
S51	S32 OR S33 OR S34 OR S35 OR S36 OR S37 OR S38 OR S39 OR S40 OR S41 OR S42 OR S43 OR S44 OR S45 OR S46 OR S47 OR S48 OR S49 OR S50
S50	TI (ICU N1 Acquired) OR AB (ICU N1 Acquired)
S49	TI (ICU N3 Infect*) OR AB (ICU N3 Infect*)
S48	TI (Community N3 Infect*) OR AB (Community N3 Infect*)
S47	TI (Community N1 Associated) OR AB (Community N1 Associated)
S46	TI (Community N1 Acquired) OR AB (Community N1 Acquired)
S45	TI (Healthcare N1 Associated) OR AB (Healthcare N1 Associated)
S44	TI (Healthcare N1 Infect*) OR AB (Healthcare N1 Infect*)
S43	TI (Healthcare N1 Acquired) OR AB (Healthcare N1 Acquired)
S42	TI (Intrahospital N1 Acquired) OR AB (Intrahospital N1 Acquired)
S41	TI (Intrahospital N1 Associated) OR AB (Intrahospital N1 Associated)

#	Query
S40	TI (Intrahospital N1 Infect*) OR AB (Intrahospital N1 Infect*)
S39	TI (Hospital* N1 Infect*) OR AB (Hospital* N1 Infect*)
S38	TI (Hospital* N1 Associated) OR AB (Hospital* N1 Associated)
S37	TI (Hospital* N1 Acquired) OR AB (Hospital* N1 Acquired)
S36	TI (Nosocomial N1 Acquired) OR AB (Nosocomial N1 Acquired)
S35	TI (Nosocomial N1 Associated) OR AB (Nosocomial N1 Associated)
S34	TI (Nosocomial N1 Infect*) OR AB (Nosocomial N1 Infect*)
S33	TI (Cross N1 Infect*) OR AB (Cross N1 Infect*)
S32	(MH “Cross Infection+”)
S31	S5 OR S6 OR S7 OR S8 OR S9 OR S10 OR S11 OR S12 OR S13 OR S14 OR S15 OR S16 OR S17 OR S18 OR S19 OR S20 OR S21 OR S22 OR S23 OR S24 OR S25 OR S26 OR S27 OR S28 OR S29 OR S30
S30	TI Enterobacter* OR AB Enterobacter*
S29	(MH “Enterobacteriaceae+”)
S28	TI Enterococc* OR AB Enterococc*
S27	(MH “Vancomycin Resistant Enterococci”)
S26	(MH “Enterococcus+”)
S25	TI (Staphylococcus N1 Aure*) OR AB (Staphylococcus N1 Aure*)
S24	(MH “Vancomycin-Resistant Staphylococcus Aureus”)
S23	(MH “Staphylococcus Aureus+”)
S22	TI Herellea* OR AB Herellea*
S21	TI Acinetobacter* OR AB Acinetobacter*
S20	(MH “Acinetobacter Infections”)
S19	TI Pseudomona* OR AB Pseudomona*
S18	(MH “Pseudomonas”)
S17	TI Jadinii OR AB Jadinii
S16	TI Monilia* OR AB Monilia*
S15	TI Candid* OR AB Candid*
S14	(MH “Candida+”)
S13	TI (Carbapenemase-Producing N3 Enterobacter*) OR AB (Carbapenemase-Producing N3 Enterobacter*)
S12	TI (Carbapenem N3 Resist*) OR AB (Carbapenem N3 Resist*)
S11	(MH “Carbapenem-Resistant Enterobacteriaceae”)
S10	TI (Cephalosporin N3 Resist*) OR AB (Cephalosporin N3 Resist*)
S9	TI (Vancomycin N3 Resist*) OR AB (Vancomycin N3 Resist*)
S8	(MH “Vancomycin Resistant Enterococci”)
S7	TI MRSA OR AB MRSA
S6	TI (Methicillin N3 Resist*) OR AB (Methicillin N3 Resist*)
S5	(MH “Methicillin-Resistant Staphylococcus Aureus”)
S4	S1 OR S2 OR S3
S3	TI AMR OR AB AMR
S2	TW Resist*
S1	(MH “Drug Resistance, Microbial+”)

Appendix Table 4. Terms used in Cochrane Library search, 29 March 2022

ID	Search
#1	MeSH descriptor: [Drug Resistance, Microbial] explode all trees
#2	Resist*:ti,ab,kw
#3	AMR:ti,ab,kw
#4	#1 OR #2 OR #3
#5	MeSH descriptor: [Methicillin-Resistant Staphylococcus aureus] explode all trees
#6	(Methicillin NEAR/3 Resist*):ti,ab,kw
#7	MRSA:ti,ab,kw
#8	MeSH descriptor: [Vancomycin-Resistant Enterococci] explode all trees
#9	(Vancomycin NEAR/3 Resist*):ti,ab,kw
#10	(Cephalosporin NEAR/3 Resist*):ti,ab,kw
#11	MeSH descriptor: [Carbapenem-Resistant Enterobacteriaceae] explode all trees
#12	(Carbapenem NEAR/3 Resist*):ti,ab,kw
#13	(Carbapenemase-Producing NEAR/3 Enterobacter*):ti,ab,kw
#14	MeSH descriptor: [Candida] explode all trees
#15	Candid*:ti,ab,kw
#16	Monilia*:ti,ab,kw
#17	Jadinii:ti,ab,kw
#18	MeSH descriptor: [Pseudomonas] explode all trees
#19	Pseudomona*:ti,ab,kw
#20	MeSH descriptor: [Acinetobacter] explode all trees
#21	Acinetobacter*:ti,ab,kw

ID	Search
#22	Herellea*:ti,ab,kw
#23	MeSH descriptor: [Staphylococcus aureus] explode all trees
#24	(Staphylococcus NEAR/1 Aure*):ti,ab,kw
#25	MeSH descriptor: [Enterococcus] explode all trees
#26	Enterococc*:ti,ab,kw
#27	MeSH descriptor: [Enterobacteriaceae] explode all trees
#28	Enterobacter*:ti,ab,kw
#29	#5 OR #6 OR #7 OR #8 OR #9 OR #10 OR #11 OR #12 OR #13 OR #14 OR #15 OR #16 OR #17 OR #18 OR #19 OR #20 OR #21 OR #22 OR #23 OR #24 OR #25 OR #26 OR #27 OR #28
#30	MeSH descriptor: [Cross Infection] explode all trees
#31	(Cross NEAR/1 Infect*):ti,ab,kw
#32	(Nosocomial NEAR/3 Infect*):ti,ab,kw
#33	(Nosocomial NEAR/3 Associated):ti,ab,kw
#34	(Nosocomial NEAR/3 Acquired):ti,ab,kw
#35	(Hospital* NEAR/3 Acquired):ti,ab,kw
#36	(Hospital* NEAR/3 Associated):ti,ab,kw
#37	(Hospital* NEAR/3 Infect*):ti,ab,kw
#38	(Intrahospital* NEAR/3 Infect*):ti,ab,kw
#39	(Intrahospital* NEAR/3 Associated):ti,ab,kw
#40	(Intrahospital* NEAR/3 Acquired):ti,ab,kw
#41	(Healthcare NEAR/3 Acquired):ti,ab,kw
#42	(Healthcare NEAR/3 Associated):ti,ab,kw
#43	(Healthcare NEAR/3 Infect*):ti,ab,kw
#44	(Community NEAR/3 Acquired):ti,ab,kw
#45	(Community NEAR/3 Associated):ti,ab,kw
#46	(Community NEAR/3 Infect*):ti,ab,kw
#47	(ICU NEAR/3 Acquired):ti,ab,kw
#48	(ICU NEAR/3 Associated):ti,ab,kw
#49	(ICU NEAR/3 Infect*):ti,ab,kw
#50	#30 OR #31 OR #32 OR #33 OR #34 OR #35 OR #36 OR #37 OR #38 OR #39 OR #40 OR #41 OR #42 OR #43 OR #44 OR #45 OR #46 OR #47 OR #48 OR #49
#51	MeSH descriptor: [Mortality] explode all trees
#52	Mortalit*:ti,ab,kw
#53	(Death NEAR/1 Rate*):ti,ab,kw
#54	(Fatality NEAR/1 Rate*):ti,ab,kw
#55	MeSH descriptor: [Quality-Adjusted Life Years] explode all trees
#56	(Quality NEAR/1 Adjusted):ti,ab,kw
#57	QALY*:ti,ab,kw
#58	(Adjusted NEAR/1 Life):ti,ab,kw
#59	(Disability NEAR/1 Adjusted):ti,ab,kw
#60	DALY*:ti,ab,kw
#61	MeSH descriptor: [Length of Stay] explode all trees
#62	(Stay NEAR/3 Length*):ti,ab,kw
#63	(Hospital* NEAR/3 Stay*):ti,ab,kw
#64	#51 OR #52 OR #53 OR #54 OR #55 OR #56 OR #57 OR #58 OR #59 OR #60 OR #61 OR #62 OR #63
#65	MeSH descriptor: [Latin America] explode all trees
#66	MeSH descriptor: [Central America] explode all trees
#67	MeSH descriptor: [South America] explode all trees
#68	MeSH descriptor: [Caribbean Region] explode all trees
#69	MeSH descriptor: [West Indies] explode all trees
#70	MeSH descriptor: [Indians, South American] explode all trees
#71	MeSH descriptor: [Indians, Central American] explode all trees
#72	MeSH descriptor: [Mexico] explode all trees
#73	MeSH descriptor: [Puerto Rico] explode all trees
#74	(Latin NEAR/1 America*) OR Latinamerica* OR Latinoamerica* OR Latin* OR Hispanic Americans OR Iberoamerica* OR (Ibero NEAR/1 Americ*) OR Panamerican* OR (Central NEAR/1 America*) OR Centroamerica* OR Mesoamerica* OR (Meso NEAR/1 America*) OR (Middle NEAR/1 America*) OR (South NEAR/1 America*) OR Southamerica* OR Sudamerica* OR (America NEAR/1 Sur) OR Caribbean OR Caribe* OR (West NEAR/1 Indi*) OR Antill* OR Amerindian* OR Indians OR (American NEAR/1 Indian*) OR (Native NEAR/1 America*) OR Patagoni* OR Andes OR Andean* OR Amazon* OR Argentin* OR Bolivia* OR Brazil* OR Brasil* Colombia* OR Colombia* OR Colombia OR Chile* OR Ecuador* OR Guiana* OR Guyan* OR Guyan* OR Paraguay* OR Paraguay* OR Peru* OR Surinam* OR Surinam* OR Uruguay* OR Venez* OR Belize* OR (Costa NEAR/1 Ric*) OR Costarric* OR Costaric* OR Costa Ric* OR Costarric* OR Salvador* OR Salvador* OR Guatemal* OR Guatemal* OR Guatemala OR Hondur* OR Nicaragu* OR Panam* OR Mexic* OR Cuba* OR Dominic* OR Dominic* OR Haiti* OR Jamaic* OR (Puerto NEAR/1 Ric*) OR Puertorric* OR Puertoric*
#75	#65 OR #66 OR #67 OR #68 OR #69 OR #70 OR #71 OR #72 OR #73 OR #74
#76	#4 AND #29 AND #50 AND #64 AND #74 with Publication Year from 2000 to 2022, with Cochrane Library publication date Between Jan 2000 and Mar 2022, in Trials

Appendix Table 5. Terms used in LILACS (BVS Eng) search, 29 March 2022

Database:	LILACS
Search on:	(MH Drug Resistance, Microbial OR Resist$ OR AMR OR RAM) AND (MH Methicillin-Resistant Staphylococcus Aureus OR Methicillin OR Meticilin$ OR MRSA OR MH Vancomycin-Resistant Enterococci OR Vancomycin OR Vancomicin$ OR Cephalosporin OR Cefalosporin$ OR MH Carbapenem-Resistant Enterobacteriaceae OR Carbapenem OR ((Carbapenemas$) AND (Producing OR Produtor$ OR Productor$)) OR MH Candida OR Candid$ OR Monilia$ OR Jadinii OR MH Pseudomonas OR Pseudomona$ OR MH Acinetobacter OR Acinetobacter$ OR Herellea$ OR MH Staphylococcus Aureus OR Staphylococcus OR Estafilococ$ OR MH Enterococcus OR Enterococ$ OR Enterococc$ OR MH Enterobacteriaceae OR Enterobacter$) AND (MH Cross Infection OR ((Cross OR Cruzad$ OR Nosocomial OR Hospital$ OR Intrahospital$ OR Healthcare OR Community OR Comunidad$ OR ICU) AND (Acquired OR Adquirid$ OR Associated OR Asociad$ OR Associad$ OR Infect$ OR Infecc$))) AND (MH Mortality OR Mortalit$ OR Mortalidad$ OR ((Death OR Muerte$ OR Morte$) AND (Rate$ OR Tasa OR Taxa$)) OR MH Quality-Adjusted Life Years OR ((Quality OR Disability) AND (Adjusted)) OR QALY$ OR DALY$ OR MH Length of Stay OR Estadía$ OR Stay$) [Words] and 2000 OR 2001 OR 2002 OR 2003 OR 2004 OR 2005 OR 2006 OR 2007 OR 2008 OR 2009 OR 2010 OR 2011 OR 2012 OR 2013 OR 2014 OR 2015 OR 2016 OR 2017 OR 2018 OR 2019 OR 2020 OR 2021 OR 2022 [Country, year publication]

Grey literature sources

· IDWEEK – Infectious Diseases Society of America (IDSA) – USA (https://idweek.org/)

· European Congress of Clinical Microbiology and Infection Diseases (ECCMID) – Europa (https://www.eccmid.org/)

· Society of Healthcare Epidemiology of America (SHEA) – USA (https://shea-online.org/)

· International Conference on Prevention & Infection Control (ICPIC) – Suiza (https://www.icpic.com/)

· Asociación Panamericana de Infectología (API) – Latinoamérica (https://www.apiinfectologia.org/)

· Asociación Latinoamericana para el Control de Infecciones (ASLACI) – Latinoamérica (https://www.aslaci.org/)

· Sociedad Argentina de Infectología (SADI) – Argentina (https://sadi.org.ar)

· Sociedad Peruana de Enfermedades Infecciosas y Tropicales – Perú (https://speit.org.pe/)

· Sociedad de Enfermedades Infecciosas de Panamá (SEIP) – Panamá (https://seipma.com)

· Asociación Centroamericana y del Caribe de Infectología (ACENCAI) / Asociación Guatemalteca de Enfermedades Infecciosas (AGEI) – Guatemala (https://asociacionageiorg.wordpress.com/)

· Sociedad de Infectología Clínica del Uruguay – Uruguay (www.infectologia.edu.uy)

· Sociedad Chilena de Infectología – Chile (http://www.sochinf.cl/)

· Sociedade Brasileira de Infectologia – Brasil (https://infectologia.org.br/)

https://jic-abih.com.br/index.php/jic/issue/view/48

https://jic-abih.com.br/index.php/jic/issue/view/Suplemento%201

https://jic-abih.com.br/index.php/jic/issue/view/35

https://jic-abih.com.br/index.php/jic/issue/view/33

https://jic-abih.com.br/index.php/jic/issue/view/29

https://jic-abih.com.br/index.php/jic/issue/view/26

https://jic-abih.com.br/index.php/jic/issue/view/17

https://jic-abih.com.br/index.php/jic/issue/view/JIC%20Vol%202%20Numero%201

https://jic-abih.com.br/index.php/jic/issue/view/9

https://infectologia.org.br/atualizacao/publicacoes-da-sbi/

Appendix Table 6. List of studies excluded at full-text screening stage

Study author and publication year	Reason for exclusion
Barrero et al (2014) (*1*)	Duplicate
CartaxoSalgado et al (2011) (*2*)	Wrong outcome
Lopez Luis et al (2019) (*3*)	Duplicate
Lucena et al (2014) (*4*)	Wrong patient population
Fiorentin Vandresen et al (2021) (*5*)	Wrong patient population
Furtado et al (2009) (*6*)	Wrong patient population
Mano et al (2021) (*7*)	Wrong patient population
Pinheiro et al (2008) (*8*)	Wrong patient population
Michelud (2021) (*9*)	Wrong outcome
Calvancanti Dantas et al (2014) (*10*)	Duplicate
Freire et al (2015) (*11*)	Wrong patient population
Bento Talizin et al (2020) (*12*)	Wrong outcome
Cassettari et al (2012) (*13*)	Wrong patient population
Cezario et al (2009) (*14*)	Wrong outcome
Cusmano et al (2013) (*15*)	Wrong patient population
Pinoni et al (2019) (*16*)	Wrong patient population
Superti et al (2009) (*17*)	Wrong outcome
Romi et al (2017) (*18*)	Wrong patient population
da Silva et al (2020) (*19*)	Wrong patient population

Appendix Table 7. Risk of Bias Assessment for Cohort Studies—Cross-Sectional studies*

	Evaluation														
Study	**1**	**2**	**3**	**4**	**5**	**6**	**7**	**8**	**9**	**10**	**11**	**12**	**13**	**14**	**Final**
Arnoni et al (2007) (*20*)	Yes	Yes	CD	Yes	No	Yes	Yes	NA	Yes	NA	Yes	NA	NA	No	POOR
Bellísimo-Rodrigues et al (2006) (*21*)	Yes	Yes	CD	Yes	No	Yes	Yes	NA	Yes	NA	Yes	NA	NA	Yes	FAIR
Bello-Chavolla et al (2018) (*22*)	Yes	Yes	Yes	Yes	No	Yes	Yes	NA	Yes	NA	Yes	NA	NR	Yes	LOW-RISK
Quillici et al (2021) (*23*)	Yes	Yes	CD	Yes	No	Yes	Yes	NA	Yes	NA	Yes	NA	NA	Yes	FAIR
Blot et al (2019) (*24*)	Yes	Yes	Yes	Yes	Yes	Yes	Yes	NA	Yes	NA	Yes	NA	Yes	Yes	LOW-RISK
Bravo et al (2017) (*25*)	Yes	No	CD	NR	No	Yes	Yes	NA	NR	NA	Yes	NA	NA	No	POOR
Caceres et al (2020) (*26*)	Yes	Yes	CD	Yes	No	Yes	Yes	NA	Yes	NA	Yes	NA	NA	No	POOR
Carneiro et al (2012) (*27*)	Yes	Yes	CD	Yes	No	Yes	Yes	NA	Yes	NA	Yes	NA	NA	Yes	FAIR
Cassettari et al (2005) (*28*)	Yes	Yes	Yes	Yes	No	Yes	Yes	NA	Yes	NA	Yes	NA	NA	No	FAIR
Castillo et al (2012) (*29*)	Yes	Yes	Yes	Yes	Yes	Yes	Yes	NA	Yes	NA	Yes	NA	NA	Yes	LOW-RISK
Copaja-Corzo et al (2020) (*30*)	Yes	Yes	Yes	Yes	Yes	Yes	Yes	NA	Yes	NA	Yes	NA	NA	Yes	LOW-RISK
Cornejo-Juarez et al (2016) (*31*)	Yes	Yes	Yes	Yes	No	Yes	Yes	NA	Yes	NA	Yes	NA	NA	No	FAIR
Cornejo-Juarez et al (2015) (*32*)	Yes	Yes	Yes	No	No	Yes	Yes	NA	Yes	NA	Yes	NA	NA	No	FAIR
Correa et al (2013) (*33*)	Yes	Yes	No	Yes	No	Yes	Yes	NA	Yes	NA	Yes	NA	NA	Yes	FAIR
da Silva et al (2021) (*34*)	Yes	Yes	Yes	Yes	No	Yes	Yes	NA	Yes	NA	Yes	NA	NA	Yes	LOW-RISK
Oliveira da Silva et al (2021) (*35*)	Yes	Yes	CD	Yes	No	Yes	Yes	NA	Yes	NA	CD	NA	NA	No	POOR
Castro-Lima et al (2019) (*36*)	Yes	Yes	Yes	Yes	No	Yes	Yes	NA	Yes	NA	Yes	NA	NA	Yes	LOW-RISK
Matos et al (2016) (*37*)	Yes	Yes	CD	Yes	No	Yes	Yes	NA	Yes	NA	Yes	NA	NA	Yes	FAIR
Costa et al (2015) (*38*)	Yes	Yes	Yes	Yes	No	Yes	Yes	NA	Yes	NA	Yes	NA	NA	Yes	FAIR
de Vedia et al (2017) (*39*)	Yes	Yes	Yes	Yes	No	Yes	Yes	NA	Yes	NA	No	NA	NA	No	POOR
Ducanteizelier et al (2017) (*40*)	Yes	Yes	CD	CD	No	Yes	Yes	NA	CD	NA	Yes	NA	NA	No	POOR
Echeverri-Toro et al (2012) (*41*)	Yes	Yes	Yes	Yes	No	Yes	Yes	NA	Yes	NA	Yes	NA	No	Yes	LOW-RISK
Gañete et al (2021) (*42*)	Yes	Yes	Yes	Yes	No	Yes	Yes	NA	Yes	NA	Yes	NA	NA	No	FAIR
Gentile et al (2018) (*43*)	Yes	Yes	Yes	Yes	No	NA	NA	NA	Yes	NA	Yes	NA	NA	No	FAIR
Gomes et al (2006) (*44*)	Yes	Yes	Yes	Yes	Yes	Yes	Yes	NA	Yes	NA	Yes	NA	NA	Yes	LOW-RISK
González et al (2014) (*45*)	Yes	Yes	Yes	Yes	No	Yes	Yes	NA	Yes	NA	Yes	NA	NA	Yes	LOW-RISK
Guilarde et al (2006) (*46*)	Yes	Yes	CD	Yes	No	Yes	Yes	NA	Yes	NA	Yes	NA	NA	Yes	FAIR
Herrera et al (2021) (*47*)	Yes	Yes	CD	Yes	No	Yes	Yes	NA	Yes	NA	Yes	NA	CD	Yes	LOW-RISK
Islas-Muñoz et al (2018) (*48*)	Yes	Yes	Yes	Yes	No	Yes	Yes	NA	Yes	NA	Yes	NA	NA	Yes	LOW-RISK
Kallel et al (2020) (*49*)	Yes	Yes	Yes	Yes	No	Yes	Yes	NA	Yes	NA	Yes	NA	NA	Yes	LOW-RISK
Karve et al (2018) (*50*)	Yes	Yes	CD	Yes	No	Yes	Yes	NA	Yes	NA	Yes	NA	NA	Yes	FAIR
Lemos et al (2014) (*51*)	Yes	Yes	Yes	Yes	No	Yes	Yes	NA	Yes	NA	Yes	NA	NA	Yes	LOW-RISK

Study	Evaluation 1	2	3	4	5	6	7	8	9	10	11	12	13	14	Final
Lipari et al (2021) (*52*)	Yes	Yes	CD	No	No	Yes	Yes	NA	Yes	NA	Yes	NA	NA	No	POOR
Lopez Luis et al (2019) (*3*)	Yes	Yes	CD	CD	No	Yes	Yes	NA	Yes	NA	Yes	NA	NA	Yes	FAIR
Marra et al (2006) (*53*)	Yes	Yes	Yes	Yes	No	Yes	Yes	NA	Yes	NA	Yes	NA	NA	Yes	LOW-RISK
Michelud et al (2021) (*9*)	Yes	Yes	CD	CD	No	Yes	Yes	NA	Yes	NA	Yes	NA	NA	No	POOR
Moreira et al (2008) (*54*)	Yes	Yes	Yes	Yes	No	Yes	Yes	NA	Yes	NA	Yes	NA	NA	No	FAIR
Nassar et al (2021) (*55*)	Yes	Yes	Yes	No	No	Yes	Yes	NA	Yes	NA	Yes	NA	NA	No	FAIR
Naves et al (2012) (*56*)	Yes	Yes	No	Yes	No	Yes	Yes	NA	Yes	NA	Yes	NA	NA	Yes	FAIR
Neves et al (2017) (*57*)	Yes	Yes	Yes	Yes	No	Yes	Yes	NA	Yes	NA	Yes	NA	NA	No	FAIR
Patermina-de la Ossa et al (2018) (*58*)	Yes	Yes	Yes	Yes	No	Yes	Yes	NA	Yes	NA	Yes	NA	NA	No	FAIR
Pinhati et al (2016)	Yes	Yes	Yes	Yes	No	NA	NA	NA	Yes	NA	Yes	NA	NA	No	FAIR
Ponce de León et al (2010) (*59*)	Yes	Yes	Yes	Yes	No	Yes	Yes	NA	Yes	NA	Yes	NA	NA	No	FAIR
Porto et al (2013) (*60*)	Yes	Yes	CD	Yes	No	Yes	Yes	NA	Yes	NA	Yes	NA	NA	No	POOR
Prata-Rocha et al (2012) (*61*)	Yes	Yes	CD	Yes	No	Yes	Yes	NA	Yes	NA	Yes	NA	NA	No	POOR
Rossi Gonçalves et al (2017) (*62*)	Yes	Yes	CD	Yes	No	Yes	Yes	NA	Yes	NA	Yes	NA	NA	No	POOR
da Silva et al (2014) (*63*)	Yes	Yes	Yes	Yes	No	Yes	Yes	NA	Yes	NA	Yes	NA	NA	No	FAIR
Seas et al (2018) (*64*)	Yes	Yes	Yes	Yes	Yes	Yes	Yes	NA	Yes	NA	Yes	NA	Yes	Yes	LOW-RISK
Seligman et al (2013) (*65*)	Yes	Yes	Yes	Yes	Yes	Yes	Yes	NA	Yes	NA	Yes	NA	NA	Yes	LOW-RISK
Tuon et al (2012) (*66*)	Yes	Yes	Yes	Yes	No	Yes	Yes	NA	Yes	NA	Yes	NA	NA	No	FAIR
Valderrama et al (2016) (*67*)	Yes	Yes	Yes	Yes	Yes	Yes	Yes	NA	Yes	NA	Yes	NA	NA	No	FAIR
Zavascki et al (2006) (*68*)	Yes	Yes	Yes	Yes	No	Yes	Yes	NA	Yes	NA	Yes	NA	Yes	Yes	LOW-RISK

*CD, cannot be determined; NA, not applicable; NR, not reported. Assessment questions 1–14 appear below.

1. Was the study question or research objective clearly specified?

2. Was the study population clearly specified and defined?

3. Did at least 50% of eligible subjects take part?

4. Were all subjects screened or recruited from the same population or from similar populations (including the same period of time)? Were inclusion and exclusion criteria to take part in the study pre-specified and applied consistently to all participants?

5. Was rationale for sample size, power description or variance and effect estimations provided?

6. For analysis of this study, were the exposures of interest measured before results?

7. Was the follow-up period enough for one to reasonably expect to observe an association between exposure and result, if any?

8. For exposures that may var.y in terms of amount or level, did the study examine different levels of exposure relative to the result (e.g., categories of exposure or exposure measured as a continuous variable)?

9. Were measures of exposure (independent variables) clearly defined, valid, reliable and consistently implemented for all study participants?

10. Were exposures evaluated more than once over time?

11. Were measures of results (dependent variables) clearly defined, valid, reliable and consistently implemented for all study participants?

12. Were results raters blinded to participants' exposure?

13. Were lost to follow-up 20% or less after the study startup?

14. Were potential confounding variables key due to their impact on the exposure(s)-result(s) ratio measured and statistically adjusted?

Appendix Table 8. Risk of Bias Assessment for Case-Control studies

	Evaluation*												
Study	**1**	**2**	**3**	**4**	**5**	**6**	**7**	**8**	**9**	**10**	**11**	**12**	**Final**
Araya et al (2019) (*69*)	Yes	Yes	No	Yes	Yes	No	NA	NA	Yes	Yes	CD	No	POOR
de Oliveira Conterno et al (2002) (*70*)	Yes	Yes	No	Yes	Yes	Yes	CD	CD	Yes	Yes	No	Yes	LOW-RISK

*CD. cannot be determined; NA, not applicable; NR, not reported. Assessment questions 1–12 appear below.

1. Was the research question or objective in this paper clearly stated and appropriate?

2. Was the study population clearly specified and defined?

3. Did the authors include a sample size justification?

4. Were controls selected or recruited from the same or similar population that gave rise to the cases (including the same timeframe)?

5. Were the definitions, inclusion and exclusion criteria, algorithms or processes used to identify or select cases and controls valid, reliable, and implemented consistently across all study participants?

6. Were the cases clearly defined and differentiated from controls?

7. If less than 100 percent of eligible cases and/or controls were selected for the study, were the cases and/or controls randomly selected from those eligible?

8. Was there use of concurrent controls?

9. Were the investigators able to confirm that the exposure/risk occurred before the development of the condition or event that defined a participant as a case?

10. Were the measures of exposure/risk clearly defined, valid, reliable, and implemented consistently (including the same time period) across all study participants?

11. Were the assessors of exposure/risk blinded to the case or control status of participants?

12. Were key potential confounding variables measured and adjusted statistically in the analyses? If matching was used, did the investigators account for matching during study analysis?

Appendix Table 9. Additional characteristics of included studies*

Study	Site of infection	Charlson Comorbidity Index	Severity Score	Empiric treatment appropriateness	Length of hospital stay (days) R	Length of hospital stay (days) S
Bravo et al (2017) (*25*)	CLABSI: 17 (23.9%); BSI: 14 (19.7%); SSTI: 9 (12.7%); SSI: 5 (7%); Cardiac: 3 (4.2%)	NR	NR	all: 61 (86%)	NR	NR
de Vedia et al (2017) (*39*)	SSTI: 37 (46.8%); Pneumonia: 62 (78.5%)†	NR	NR	NR	NR	NR
Ducanteizelier et al (2017) (*40*)	UTI: 35 (32.7%); P-IAI: 25 (23.4%); Pneumonia: 14 (13.1%); BSI: 12 (11.2%); SSTI: 10 (9.3%)	NR	NR	R: 4 (10.5%); S: 54 (79%)	NR	NR
Gañete et al (2021) (*42*)	BSI: 71 (71%); CLABSI: 18 (18%); SSTI: (9%); B&J: (4%); Cardiac: (1.1%)†	NR	NR	NR	NR	NR
Gentile et al (2018) (*43*)	NR	NR	NR	NR	NR	NR
Herrera et al (2021) (*47*)	CLABSI: 358 (28%); P-IAI: 214 (16.8%); Pneumonia: 118 (9.2%); Other infections: 916 (71.7%)†	NR	NR	NR	NR	NR
Lipari et al (2021) (*52*)	NR	NR	NR	NR	NR	NR
Michelud et al (2021) (*9*)	UTI: 263 (54%); P-IAI: 111 (22.8%)	NR	NR	all: 345 (70.8%); R: 19 (24.6%)	12 (16.2)‡	8 (9)‡
Arnoni et al (2007) (*20*)	BSI: 92 (100%)	NR	NR	NR	NR	NR
Bellísimo-Rodrigues et al (2006) (*21*)	BSI: 33 (31.7%); Pneu: 31 (29.8%); SSTI: 23 (22.1%); P-IAI: 7 (6.7%); UTI: 5 (4.8%)	NR	NR	NR	25.1‡	23.7‡
Quillici et al (2021) (*23*)	BSI: 270 (100%)	≥3: 86 (31.8%)	NR	all: 79 (29.3%); R: 27 (15.1%)	NR	NR
Carneiro et al (2012) (*27*)	BSI: 132 (100%)	NR	NR	NR	NR	NR
Cassettari et al (2005) (*28*)	BSI: 41 (25.2%); CLABSI: 33 (20.2%); Pneumonia: 26 (16%); SSI: 20 (12.3%); SSTI: 10 (6.1%)	NR	NR	all: 149 (91.4%)	NR	NR
Correa et al (2013) (*33*)	NR	≥3: 23 (38%)	APACHE II: R: 22.1‡; S: 16.4‡	NR	20‡	32‡
da Silva et al (2021) (*34*)	BSI: 257 (72%); CLABSI: 123 (34.5%); Unknown: 63 (17.6%); P-IAI: 23 (6.4%); Other infections: 16 (4.5%)†	4.48 (0–15)§	Pitt Score ≥4: 112 (31.4%)	all: 225 (63.2%)	NR	NR
Oliveira da Silva et al (2021) (*35*)	NR	2.5 (1–3)§	SAPS III: R: 53 (46–61)§ S: 52 (47–59)§	NR	63 (27–145)§	34.5 (18–74)§
Castro-Lima et al (2019) (*36*)	CLABSI: 42 (55.3%); VAP: 34 (44.7%); CAUTI: 8 (10.5%)†	NR	SOFA: 6 (3–10.8)§	NR	24‡	NR
Matos et al (2016) (*37*)	BSI: 25 (46.3%); Pneumonia: 24 (44.4%); UTI: 5 (9.3%)	0–2: 28 (52%)	McCabe (Potentially fatal): R: 13 (65%); S: 11 (32.3%)	R: 0 (0%); S: 15 (44.2%)	≥30 d: 18 (90%)	≥30 d: 25 (73.5%)

Study	Site of infection	Charlson Comorbidity Index	Severity Score	Empiric treatment appropriateness	Length of hospital stay (days) R	Length of hospital stay (days) S
de Oliveira Conterno et al (2002) (*70*)	CLABSI: 55 (21.9%); Pneumonia: 45 (17.9%); Other infections: 42 (16.7%); Unknown: 90 (35.9%)	NR	NR	(1991–92) all: 94 (69%) (1995–96) all: 102 (89%)	NR	NR
Costa et al (2015) (*38*)	NR	NR	NR	R:12 (25.5%); S: 34 (63%)	8§	2§
Gomes et al (2006) (*44*)	UTI: 55 (38.5%); SSI: 27 (18.9%); BSI: 23 (16.1%); Pneumonia: 18 (12.6%); P-IAI: 14 (9.8%)	NR	NR	all: 70 (49%)	NR	NR
Guilarde et al (2006) (*46*)	CLABSI: 22 (19.8%); BSI: 18 (16.2%)	NR	NR	all: 67 (60%)	NR	NR
Karve et al (2018) (*50*)	UTI: 80 (100%)	2.4 (2.3)‡	NR	all: 21 (25.6%)	NR	NR
Marra et al (2006) (*53*)	Pneumonia: 41 (38%); P-IAI: 28 (25.9%); CLABSI:15 (13.9%); UTI: 12 (11.1%); Other infections: 6 (5.6%)	NR	McCabe (Potentially fatal): R: 21 (37.5%); S: 32 (62.5%)	R: 27 (47.8%); S: 27 (52.2%)	NR	NR
Moreira et al (2008) (*54*)	VAP: 61 (100%)	NR	NR	all: 37 (60.6%); R: 14 (48.7%); S: 16 (51.3%)	NR	NR
Nassar et al (2021) (*55*)	NR	NR	SAPS III: R: 62.3 (17.6)†; S: 56.7 (16.3)‡	NR	23.9 (14.9)‡	16.6 (11.4)‡
Naves et al (2012) (*56*)	BSI: 51 (100%)	NR	NR	all: 34 (66.6%); R: 19 (65.5%); S: 15 (68.2%)	NR	NR
Neves et al (2017) (*57*)	NR	NR	NR	NR	NR	NR
Patermina-de la Ossa et al (2018) (*58*)	SSTI: 161 (57.7%); BSI: 52 (18.6%); B&J: 35 (12.5%); Pneumonia: 26 (9.3%); CNS: 23 (8.2%)†	NR	NR	NR	29.3 (12.6–60.3)§	14 (7–31)§
Pinhati et al (2016) (*71*)	BSI: 40 (100%)	NR	APACHE II: R: 14 (3–29)§; S: 16 (3–30)§	NR	NR	NR
Porto et al (2013) (*60*)	BSI: 230 (100%)	NR	NR	NR	NR	NR
Prata-Rocha et al (2012) (*61*)	Pneumonia: 37 (50.7%); BSI: 33 (45.2%); UTI: 15 (20.5%)†	NR	NR	all: 43 (58.9%) R: 29 (59.2%); S: 14 (58.3%)	NR	NR
Rossi Gonçalves et al (2017) (*62*)	BSI: 98 (62.4%); CLABSI 21 (13.4%); Pneumonia: 27 (17.2%); UTI: 6 (3.8%)	NR	ASIS Score ≥4: all: 69 (51.6%); R: 35 (50.7%); S: 33 (50.7%)	all: 92 (69%); R: 37 (54%); S: 57 (88%)	60.01 (48.97)‡	68.86 (110.4)‡
da Silva et al (2014) (*63*)	BSI: 304 (100%)	NR	NR	NR	36 (22.5)‡	NR
Seligman et al (2013) (*65*)	HAP: 140 (100%)	NR	NR	NR	NR	NR
Tuon et al (2012) (*66*)	BSI: 77 (100%)	NR	NR	all: 77 (40.3%); R: 8 (28%); S: 23 (48%)	43 (31.7)‡	43.1 (31.2)‡

Study	Site of infection	Charlson Comorbidity Index	Severity Score	Empiric treatment appropriateness	Length of hospital stay (days) R	Length of hospital stay (days) S
Zavascki et al (2006) (*68*)	Pneumonia: 150 (50.3%); UTI: 61 (20.5%); SSTI: 47 (15.8%); P-IAI: 25 (8.4%); CLABSI: 23 (7.7%)†	3.4 (1.3–5.4)§	NR	R: 39 (45%)	NR	NR
Caceres et al (2020) (*26*)	BSI: 90 (100%)	NR	NR	NR	NR	NR
Castillo et al (2012) (*29*)	CLABSI: 139 (37.4%); Pneumonia: 56 (15.1%); SSTI: 22 (5.9%); SSI: 12 (3.2%); Unknown: 113 (30.4%)	>3: 188 (50.5%)	APACHE II: all: 15 (11–21)§; R: 15 (11–22)§; S: 15 (10–20)§	all: 247 (66.3%)	30‡	21‡
Echeverri-Toro et al (2012) (*41*)	UTI: 104 (42.8%); BSI: 54 (22.2%); Pneumonia: 33 (13.6%); B&J: 19 (7.8%); P-IAI: 17 (7%)	NR	NR	NR	>30 d: 43 51.2%)	>30 d: 50 (31.4%)
González et al (2014) (*45*)	Pneumonia: 87 (40.1%); CLABSI: 67 (30.9%); SSTI: 29 (13.4%); P-IAI: 27 (12.4%)	3 (5)‡	APACHE II: all: 14 (10)§	all: 122 (56.3%)	NR	NR
Lemos et al (2014) (*51*)	Pneumonia: 57 (34.5%); SSI: 24 (24.2%); BSI: 24 (14.5%); CLABSI: 20 (12.1%); UTI: 7 (4.2%)	NR	APACHE II: R: 12.8 (5.4)‡; S: 10.1 (4.6)‡	R: 64 (61.5%); S: 51 (83.6%)	13.2 (13.8)‡	10.1 (8.7)‡
Valderrama et al (2016) (*67*)	Pneumonia: 51 (30.4%); P-IAI: 44 (26.2%); BSI: 23 (13.7%); UTI: 17 (10.1%); CLABSI: 7 (4.2%)	NR	APACHE II: R: 14.5 (0–29)§; S: 11.5 (0–33)§	R: 22 (52.4%); S: 95 (75.4%)	General ward: 26 (3–115)§; ICU: 12.5 (0–106)§	General ward: 16 (1–161)§ ICU: 8 (0–161)§
Kallel et al (2020) (*49*)	BSI: 98 (43.9%); CLABSI: 37 (16.6%); VAP: 27 (12.1%); UTI: 15 (6.7%); SSTI: 5 (2.2%)	NR	SAPS: all: 50 (40–64)‡; R: 57 (42–75)§; S: 50 (39–63)§	all: 223 (65.1%); R: 24 (61.5%); S: 127 (69%)	37 (18–57)§	24 (15–48)§
Blot et al (2019) (*24*)	P-IAI: 364 (100%)	NR	NR	NR	NR	NR
Seas et al (2018) (*64*)	CLABSI: 378 (41.3%); BSI: 180 (19.7%); SSTI: 80 (8.7%); Pneumonia: 77 (8.4%); B&J: 60 (6.6%)	2 (1–3)‡	APACHE II: R: 0 (1–14)§; S: 0 (0–12)§	R: 232 (74.5%); S: 188 (51.9%)	ICU: 17.2 (16.6)‡; Site not specified: 39.1 (37.6)‡	ICU: 13.6 (14.8)†; Site not specified: 28.6 (31.2)‡
Bello-Chavolla et al (2018) (*22*)	CLABSI: 254 (56.4%); CAP: 34 (7.6%); HCAP: 33 (7.3%); SSTI: 28 (6.2%); P-IAI: 27 (6%)	4 (2–6)‡	NR	all: 308 (68.4%)	NR	NR
Cornejo-Juarez et al (2016) (*31*)	VAP: 34 (53.1%); CAUTI: 22 (34.4%); SSI: 13 (20.3%); CLABSI: 4 (6.2%); P-IAI: 3 (4.7%)†	NR	APACHE II: 18.9 (6.4)‡	NR	NR	NR
Cornejo-Juarez et al (2015) (*32*)	VAP: 72 (53.7%); CAUTI: 41 (30.6%); SSI: 21 (15.7%); P-IAI: 20 (14.9%); CLABSI: 5 (3.7%)†	NR	SOFA: 8.4 (3.3)†	all: 135 (56%)	16 (10)‡	12 (6)‡
Islas-Muñoz et al (2018) (*48*)	CLABSI: 156 (31.5%); UTI: 93 (18.8%); P-IAI: 82 (16.5%); BSI: 75 (15.1%); SSTI: 34 (6.9%)	>2: 245 (49.5%)	NR	all: 448 (90.3%)	NR	NR
Lopez Luis et al (2019) (*3*)	BSI: 192 (100%)	NR	NR	NR	NR	NR
Ponce de León et al (2010) (*59*)	CLABSI: 70 (40.7%); Pneumonia: 45 (26.2%); SSTI: 28 (16.3%); B&J: 10 (5.8%); Cardiac: 8 (4.7%)	NR	NR	all: 172 (82%); R: 57 (72.1%); S: 86 (90.3%)	31§	21§

Study	Site of infection	Charlson Comorbidity Index	Severity Score	Empiric treatment appropriateness	Length of hospital stay (days) R	Length of hospital stay (days) S
Araya et al (2019) (*69*)	BSI: 50 (46.7%); CAP: 50 (46.7%); B&J: 30 (28%); Cardiac: 4 (3.7%)†	NR	NR	NR	NR	NR
Copaja-Corzo et al (2021) (*30*)	VAP: 37 (29.8%); CLABSI: 10 (8.1%); CAUTI: 3 (2.4%)	NR	NR	NR	NR	NR

*Values are no. (%). B&J: Infections of bone, joints, and related organs; BSI: Bloodstream infection; CAP: Community-acquired pneumonia; Cardiac: Endocarditis and other cardiac infections; CAUTI: Catheter-associated urinary tract infection; CLASBI: Central line-associated bloodstream infections; CNS: Meningitis and other bacterial central nervous system infections; HAP: Hospital-acquired pneumonia; HCAP: Healthcare-associated pneumonia; LOS: length of stay; NR: not reported; P-IAI: Peritoneal and intra-abdominal infections; R: resistant group; S: susceptible group; SSI: Surgical site infection; SSTI: Skin and soft tissue infections; UTI: Urinary tract infection; VAP: Ventilator-associated pneumonia.

†More than one site of infection per patient.

‡Reported as mean (SD).

§Reported as median (interquartile range or range).

Appendix Table 10. Meta-regression analysis of adjusted measures*

Variable	No. studies	No. patients (No. of R-bacteria/No. of S-bacteria)	Coefficient	95% CI	p-value from meta regression
Year	14	4472 (1877/2595)	−0.006	−0.027–0.015	0.568
Population risk	14	4472 (1877/2595)	−0.072	−0.619–0.473	0.794
Appropriate antimicrobial therapy†	8	2789 (1155/1634)	0.1072	−0.369–0.583	0.583
Resistance mechanism‡	14	4472 (1877/2595)	··	··	0.726
Multidrug resistant bacteria	4	1009 (398/611)	Ref.	··	··
Carbapenem-resistant	3	1234 (498/736)	−0.038	−0.515–0.437	0.872
Extended spectrum β-lactamase	2	236 (113/123)	0.287	−0.271–0.845	0.313
Methicillin-resistant	5	1993 (868/1125)	0.021	−0.364–0.405	0.917

*We converted all non-RR point estimates into RRs for meta-regression analysis as previously published (*72*,*73*). The table shows the meta-regression results for study and patient characteristics of an aRR for the association between resistance and lethality.
†We consider an appropriate therapy if at least one group (resistant, susceptible, or both) has more than 70% of patients who have received early appropriate empirical therapy.
‡For the analysis we considered different types of resistance mechanism as a categorical feature: multidrug resistant bacteria (as reference), Carbapenem-resistant, Extended spectrum β-lactamase and Methicillin-resistant.

Appendix Table 11. Sensitivity analysis in the association between resistance and lethality with Knapp-Hartung adjustments

Type of adjustment	Type of association	Random effects model effect estimate (95% CI)
Without Knapp-Hartung adjustment	aOR	1.93 (1.58–2.37)
With Knapp-Hartung adjustment	aOR	1.93 (1.55–2.41)
Without Knapp-Hartung adjustment	aHR	1.31 (0.93–1.83)
With Knapp-Hartung adjustment	aHR	1.31 (0.81–2.12)

*aOR, adjusted odds ratio; aHR, adjusted hazard ratio.

Appendix Table 12. Characteristics of included studies*

Study	Country	Inclusion period	Study design	Sample size	Population risk	Age group	Site of admission	Resistant germs	Quality assessment
Bravo et al (2017)	Argentina	2016–17	RC	71	Unknown	Adults	NR	MRSA	Poor
de Vedia et al (2017)	Argentina	2002–17	RC	79	High	Adults	ICU	MRSA	Poor
Ducanteizelier et al (2017)	Argentina	2016–17	RC	107	Unknown	Adults	NR	CRE	Poor
Gañete et al (2021)	Argentina	2017–18	PC	100	High	Adults	NR	MRSA	Fair
Gentile et al (2018)	Argentina	2012–14	CS	1141	Average	Pediatric	NR	MRSA	Fair
Herrera et al (2021)	Argentina	2014–20	PC	1277†	High	Adults	ICU and non-ICU	CR-GNB	Low-Risk
Lipari et al (2021)	Argentina	2015–21	RC	186	Unknown	Adults	NR	CRE	Poor
Michelud et al (2021)	Argentina	2014–18	RC	487	High	Elderly	ICU and non-ICU	ESBL-E	Poor
Arnoni et al (2007)	Brazil	2001–03	RC	85	High	Neo/Pediatric	ICU and non-ICU	MDR-GNB	Poor
Bellísimo-Rodrigues et al (2006)	Brazil	2002–03	PC	104	High	Adults	NR	ESBL-E	Fair
Quillici et al (2021)	Brazil	2012–18	RC	270	High	Adults	ICU	MDR-GNB	Fair
Carneiro et al (2012)	Brazil	2007–11	RC	132	High	Neonatal	ICU	ESBL-E	Fair
Cassettari et al (2005)	Brazil	1999	PC	163	Average	All ages	ICU and non-ICU	MRSA	Fair
Correa et al (2013)	Brazil	2006–08	RC	60	High	Adults	ICU and non-ICU	CRE	Fair
da Silva et al (2021)	Brazil	2013–15	PC	357	High	Adults	NR	MDRO	Low-Risk
Oliveira da Silva et al (2021)	Brazil	2019	RC	331	High	Adults	ICU	MDRO	Poor
Castro-Lima et al (2019)	Brazil	2013–17	RC	76	High	Adults	ICU	MDRO	Low-Risk
Matos et al (2016)	Brazil	2010–12	RC	54	High	All ages	ICU	CR-PA	Fair
de Oliveira Conterno et al (2002)	Brazil	1991–96	CC	251	High	Adults	NR	MRSA	Low-Risk
Costa et al (2015)	Brazil	2009–12	RC	76	High	Pediatric	ICU	MDR-GNB	Fair
Gomes et al (2006)	Brazil	1998	RC	143	High	Adults	ICU and non-ICU	ESBL-E	Low-Risk
Guilarde et al (2006)	Brazil	2000–01	RC	111	High	All ages	ICU and non-ICU	MRSA	Fair
Karve et al (2018)	Brazil	2013–14	RC	80	High	Adults	NR	MDR-GNB	Fair
Marra et al (2006)	Brazil	1996–01	RC	108	High	All ages	ICU and non-ICU	ESBL-E	Low-Risk
Moreira et al (2008)	Brazil	2005–07	RC	61	High	Adults	ICU	MRSA	Fair
Nassar et al (2021)	Brazil	2019–20	PC	307	High	Adults	ICU	MDRO	Fair
Naves et al (2012)	Brazil	2007–08	RC	39	Average	All ages	General ward	MRSA	Fair
Neves et al (2017)	Brazil	2011–14	RC	1261	Average	Adults	ICU and non-ICU	CRE	Fair
Patermina-de la Ossa et al (2018)	Brazil	2012–16	RC	279	Average	Pediatric	Emergency department	MRSA	Fair
Pinhati et al (2016)	Brazil	2011–12	CS	40	High	Adults	ICU	AZC	Fair
Porto et al (2013)	Brazil	2010	RC	230	High	Adults	NR	MRSA	Poor
Prata-Rocha et al (2012)	Brazil	2009–10	PC	73	High	All ages	ICU and non-ICU	CR-AB	Poor
Rossi Gonçalves et al (2017)	Brazil	2009–12	RC	157	High	All ages	ICU and non-ICU	CR-PA	Poor
Santos da Silva et al (2014)	Brazil	1998–08	RC	304	Average	All ages	ICU and non-ICU	VRE	Fair
Seligman et al (2013)	Brazil	2007–09	RC	140	High	Adults	General ward	MDR-GNB	Low-Risk
Tuon et al (2012)	Brazil	2006–09	RC	77	High	Adults	ICU and non-ICU	CR-PA	Fair
Zavascki et al (2006)	Brazil	2004–05	PC	298	High	Adults	NR	CR-PA	Low-Risk
Caceres et al (2020)	Colombia	2015–16	RC	90	High	All ages	ICU	AZC	Poor
Castillo et al (2012)	Colombia	2005–06	RC	372	High	Adults	ICU and non-ICU	MRSA	Low-Risk
Echeverri-Toro et al (2012)	Colombia	2009–10	PC	243	High	Adults	ICU and non-ICU	ESBL-E	Low-Risk
González et al (2014)	Colombia	2005–08	RC	217	High	Adults	ICU	CR-PA	Low-Risk
Lemos et al (2014)	Colombia	2006–10	PC	165	High	Adults	ICU	CR-AB	Low-Risk
Valderrama et al (2016)	Colombia	2008–14	RC	168	High	Adults	ICU and non-ICU	CR-PA	Fair
Kallel et al (2020)	French Guiana	2013–19	RC	223	High	Adults	ICU	ESBL-E	Low-Risk

Appendix Table 12. Characteristics of included studies*

Study	Country	Inclusion period	Study design	Sample size	Population risk	Age group	Site of admission	Resistant germs	Quality assessment
Blot et al (2019)	LAC‡	2016	PC	364	High	Adults	ICU	MDRO	Low-Risk
Seas et al (2018)§	LAC¶	2011–14	PC	915§	High	Adults	ICU and non-ICU	MRSA	Low-Risk
Bello-Chavolla et al (2018)	Mexico	2006–14	RC	450	High	Adults	ICU and non-ICU	MRSA	Low-Risk
Cornejo-Juarez et al (2016)	Mexico	2003–04	PC	64	High	Adults	ICU	MDRO	Fair
Cornejo-Juarez et al (2015)	Mexico	2007–11	RC	134	High	Adults	ICU	MDRO	Fair
Islas-Muñoz et al (2018)	Mexico	2016–17	PC	496	High	Adults	NR	MDRO	Low-Risk
Lopez Luis et al (2019)	Mexico	2007–17	RC	192	Unknown	NR	NR	VRE	Fair
Ponce de León et al (2010)	Mexico	2003–07	RC	172	High	Adults	ICU and non-ICU	MRSA	Fair
Araya et al (2019)	Paraguay	2010–14	CC	107	Average	Pediatric	ICU and non-ICU	MRSA	Poor
Copaja-Corzo et al (2021)	Peru	2020–21	RC	124	High	Adults	ICU	XDRO	Low-Risk

*ARC, Azol-resistant Candida spp.; CC, case-control; CR-AB, Carbapenem-resistant Acinetobacter baumannii; CRE, Carbapenem-resistant Enterobacterales; CR-GNB, Carbapenem-resistant Gram-negative bacilli; CR-PA, Carbapenem-resistant Pseudomonas aeruginosa; CS, cross-sectional; ESBL-E, extended spectrum β-lactamase producing Enterobacterales; ICU, intensive care unit; MDR-GNB, Multidrug resistant Gram-negative bacilli; MDRO, Multidrug resistant organisms; MRSA, methicilin-resistant Staphylococcus aureus; NR, not reported; PC, prospective cohort; RC, retrospective cohort; VRE, Vancomycin-resistant Enterococcus spp.; XDRO, Extremely drug-resistant organisms.
†771 out of 1277 patients were included in the final analysis.
‡The following countries were included: Argentina, Chile, Colombia, Ecuador, Jamaica, Mexico, Paraguay, Peru.
§675 out of 915 patients were included in the final analysis.
¶The following countries were included: Argentina, Chile, Brazil, Colombia, Ecuador, Guatemala, Mexico, Perú, Venezuela.

Appendix Table 13. Outcome and association measures of included studies*

Study	In-hospital lethality R (n/N)	In-hospital lethality S (n/N)	15-d lethality R (n/N)	15-d lethality S (n/N)	30-d lethality R (n/N)	30-d lethality S (n/N)	Unadjusted OR/RR/HR (95% CI)	Adjusted OR/RR/HR (95% CI)	Type of adjustment
Methicilin-resistant *Staphylococcus aureus* (MRSA)	..	..	..	..	..	..	..	..	..
Araya et al (2019)	6/42	10/65	NR	NR	NR	NR	OR: 0.91 (0.30–2.74)	NR	..
Bello-Chavolla et al (2018)	NR	NR	NR	NR	30/95	45/355	OR: 3.18 (1.86–5.42)†	HR: 2.69 (1.52–4.76)	Charlson Comorbidity Index
Blot et al (2019	NR	NR	NR	NR	2/3	0/1	..	..	..
Bravo et al (2017)	11/22	25/49	NR	NR	11/22	25/49	OR: 0.96 (0.35–2.63)†	NR	..
Cassettari et al (2005)	43/96	20/67	32/96	18/67	NR	NR	OR: 1.91 (0.99–3.69)	NR	..
Castillo et al (2012)	106/186	86/186	NR	NR	106/186	86/186	HR: 1.31 (0.96–1.79)	HR: 0.90 (0.62–1.30)	NR
da Silva et al (2021)	NR	NR	NR	NR	13/38	15/35	OR: 0.69 (0.27–1.79)†	NR	..
de Oliveira Conterno et al (2002) (Total)	NR	NR	73/159	19/92	NR	NR	OR: 3.3 (1.8–5.9)	OR: 1.80 (1.27–2.54)	Treatment received
de Oliveira Conterno et al (2002) (1991–1992)	NR	NR	44/90	9/46	NR	NR	OR: 3.93 (1.7–9.09)	NR	..
de Oliveira Conterno et al (2002) (1995–1996)	NR	NR	29/69	10/46	NR	NR	OR: 2.61 (1.12–6.10)	NR	..

Study	In-hospital lethality R (n/N)	In-hospital lethality S (n/N)	15-d lethality R (n/N)	15-d lethality S (n/N)	30-d lethality R (n/N)	30-d lethality S (n/N)	Unadjusted OR/RR/HR (95% CI)	Adjusted OR/RR/HR (95% CI)	Type of adjustment
de Vedia et al (2017)	19/50	2/29	NR	NR	NR	NR	OR: 8.27 (1.76–38.8)†	NR	..
Gañete et al (2021)‡	4/38	14/65	NR	NR	NR	NR	OR: 0.43 (0.13–1.41)†	NR	..
Gentile et al (2018)	20/904	4/237	NR	NR	NR	NR	OR: 1.32 (0.45–3.89)	NR	..
Guilarde et al (2006)	29/61	10/50	NR	NR	33/61	12/50	HR: 3.94 (1.70–9.10)	HR: 2.52 (0.96–6.60)	Age, sex and severity of underlying disease
Islas-Muñoz et al (2018)§	0	1	NR	NR	2/6	4/43	OR: 4.88 (0.67–35.48)	NR	..
Moreira et al (2008)	11/29	8/32	NR	NR	NR	NR	OR: 1.83 (0.54–6.34)	NR	..
Naves et al (2012)‡	17/29	7/22	NR	NR	NR	NR	OR: 3.0 (0.95–9.71)	OR: 0.5 (0.11–2.24)	¶
Patermina-de la Ossa et al (2018)‡	8/120	5/159	NR	NR	NR	NR	OR: 2.20 (0.70–6.90)	NR	..
Ponce de León et al (2010)	29/79	32/93	NR	NR	17/79	20/93	In-hospital mortality: OR: 0.41 (0.14–1.22) 30 d mortality: OR: 1.00 (0.48–2.07)	NR	..
Porto et al (2013)	10/61	20/169	NR	NR	NR	NR	OR: 1.46 (0.64–3.33)	NR	..
Seas et al (2018)#	NR	NR	NR	NR	132/367	123/442	RR: 1.28 (1.06–1.55)	RR: 1.09 (0.96–1.22)	Age, Charlson comorbidity score, Pittsbirgh bacteraemia score, severity of sepsis, hospital clustering, previous surgery
Seligman et al (2013)	23/38	7/14	NR	NR	NR	NR	OR: 1.53 (0.45–5.26)†	NR	..
Vancomycin-resistant *Enterococcus* spp. (VRE)	..	..	..	..	..	..	..	..	..
Blot et al (2019)	NR	NR	NR	NR	5/11	6/28	OR: 3.06 (0.69–13.57)†	NR	..
da Silva et al (2021)	NR	NR	NR	NR	3/5	7/19	OR: 2.57 (0.34–19.33)†	NR	..
Lopez Luis et al (2019)	NR	NR	NR	NR	64/107	20/85	OR: 4.84 (2.57–9-11)	NR	..
Santos da Silva et al (2014)	23/30	NR/274	NR	NR	NR	NR	OR: 2.73 (1.09–7.78)	NR	..

Study	In-hospital lethality R (n/N)	In-hospital lethality S (n/N)	15-d lethality R (n/N)	15-d lethality S (n/N)	30-d lethality R (n/N)	30-d lethality S (n/N)	Unadjusted OR/RR/HR (95% CI)	Adjusted OR/RR/HR (95% CI)	Type of adjustment
Extended spectrum β-lactamase producing Enterobacterales (ESBL-E)	..	..	..	..	..	..	..	..	..
Bellísimo-Rodrigues et al (2006)	26/47	21/57	16/47	11/57	NR	NR	OR: 2.12 (0.97–4.67)†	OR: 2.3 (0.7–7.5)	**
Blot et al (2019)	NR	NR	NR	NR	24/59	25/81	OR: 1.54 (0.76–3.10)†	NR	..
Carneiro et al (2012)	27/66	16/66	NR	NR	25/66	8/66	OR: 2.16 (1.03–4.57)	OR: 3.47	NR
da Silva et al (2021)	NR	NR	NR	NR	15/54	53/138	OR: 0.62 (0.31–1.23)†	NR	..
Echeverri-Toro et al (2012)	NR	NR	NR	NR	12/84	30/161	OR: 0.72 (0.35–1.49)	NR	..
Gomes et al (2006)	NR	NR	NR	NR	14/68††	16/75††	RR: 0.97 (0.51–1.83)	NR	..
Islas-Muñoz et al (2018)	13	12	NR	NR	37/123	35/148	OR: 1.39 (0.81–2.39)	NR	..
Kallel et al (2020)	NR	NR	NR	NR	12/39‡‡	45/184‡‡	OR: 1.37 (0.64–2.93)	NR	..
Marra et al (2006)	NR	NR	18/56	8/52	NR	NR	OR: 2.61 (1.02–6.66)	NR	..
Michelud et al (2021)	17/77	76/410	NR	NR	NR	NR	RR: 1.19 (0.74–1.89)	NR	..
Carbapenem-resistant Enterobacterales (CRE)	..	..	..	..	..	..	..	..	..
Blot et al (2019)	NR	NR	NR	NR	8/20	41/120	OR: 1.28 (0.49–3.39)†	NR	..
Correa et al (2013)	10/20	11/40	NR	NR	NR	NR	OR: 2.64 (0.86–8.07)	NR	..
da Silva et al (2021)	NR	NR	NR	NR	16/25	53/138	OR: 2.85 (1.18–6.91)†	NR	..
Ducanteizelier et al (2017)	8/40	8/68	NR	NR	NR	NR	OR: 1.88 (0.64–5.47)†	NR	..
Lipari et al (2021)	NR	NR	NR	NR	34/93	14/93	OR: 3.3 (1.6–6.6)	NR	..
Neves et al (2017)	49/97	268/1164	NR	NR	NR	NR	OR: 3.41 (2.24–5.20)†	NR	..
Carbapenem-resistant *Pseudomonas aeruginosa* (CR-PA)	..	..	..	..	..	..	..	..	..
Blot et al (2019)	NR	NR	NR	NR	2/3	3/12	OR: 6.00 (0.39–92.28)†	NR	..
da Silva et al (2021)	NR	NR	NR	NR	13/19	3/21	OR: 10.83 (2.25–52.2)†	NR	..
González et al (2014)	NR	NR	NR	NR	43/70	70/147	OR: 2.35 (1.1–5.1)	NR	..
Islas- Muñoz et al (2018)§§	4	2	NR	NR	6/9	5/34	OR: 11.6 (2.16–62.22)	NR	..

Study	In-hospital lethality R (n/N)	In-hospital lethality S (n/N)	15-d lethality R (n/N)	15-d lethality S (n/N)	30-d lethality R (n/N)	30-d lethality S (n/N)	Unadjusted OR/RR/HR (95% CI)	Adjusted OR/RR/HR (95% CI)	Type of adjustment
Rossi Gonçalves et al (2017)	48/69	32/65	NR	NR	NR	NR	OR: 2.35 (1.16–4.78)	NR	..
Tuon et al (2012)	NR	NR	NR	NR	13/29	26/48	OR: 0.68 (0.27–1.73)	NR	..
Valderrama et al (2016)	24/42	45/126	NR	NR	NR	NR	OR: 2.40 (1.18–4.89)†	NR	..
Zavascki et al (2006)	44/86	68/212	NR	NR	NR	NR	RR: 1.60 (1.20–2-12); HR: 1.55 (1.06–2.27)	HR: 1.07 (0.72–1.60)	Age, severe sepsis or septic shock, appropriate therapy, surgical procedure
Carbapenem-resistant *Acinetobacter baumannii* (CR-AB)	..	..	..	..	..	..	..	..	..
Blot et al (2019)	NR	NR	NR	NR	2/7	0/4	..	..	..
da Silva et al (2021)	NR	NR	NR	NR	30/50	3/11	OR: 4 (0.95–16.9)†	NR	..
Lemos et al (2014)	NR	NR	NR	NR	42/104	13/61	HR: 2.12 (1.14–3.95)	HR: 1.45 (0.74–2.87)	Age, gender, APACHE II score, number of diagnoses, and inappropriate empirical treatment
Azol-resistant *Candida* spp.	..	..	..	..	..	..	..	..	..
Caceres et al (2020)	23/40	26/50	NR	NR	17/40	20/50	In-hospital mortality: OR: 1.41 (0.59–3.34) 30 d mortality: OR: 1.25 (0.52–3.01)	NR	..
Pinhati et al (2016)	NR	NR	NR	NR	9/21	9/19	OR: 0.90 (0.30–2.75)	NR	..
Multidrug-resistant organisms (MDRO)	..	..	..	..	..	..	..	..	..
Arnoni et al (2007)‡	NR	NR	NR	NR	22/44	12/48	OR: 3.00 (1.24–7.24)†	NR	..
Quillici et al (2021) (Total)	NR	NR	NR	NR	99/180	46/90	OR: 1.17 (0.70–1.94)†	NR	..
Quillici et al (2021)¶¶	NR	NR	NR	NR	39/60	12/28	OR: 2.48 (0.99–6.20)	OR: 1.7 (0.8–3.5)	NR
Blot et al (2019) (Total)##	NR	NR	NR	NR	43/103	75/246	OR: 1.63 (1.01–2.63)†	OR: 1.51 (0.85–2.69)	Intra-abdominal infection with or without anatomic barrier disruption, age, liver disease, CHF, source

Study	In-hospital lethality R (n/N)	In-hospital lethality S (n/N)	15-d lethality R (n/N)	15-d lethality S (n/N)	30-d lethality R (n/N)	30-d lethality S (n/N)	Unadjusted OR/RR/HR (95% CI)	Adjusted OR/RR/HR (95% CI)	Type of adjustment
									control achieved at day 7
Cornejo-Juarez et al (2016)	NR	NR	NR	NR	12/38	13/26	OR: 0.46 (0.16–1.29)†	NR	..
Cornejo-Juarez et al (2015)‡	51/105	7/51	NR	NR	NR	NR	OR: 17.5 (7.49–41.1)†	NR	..
Oliveira da Silva et al (2021)	59/113	89/124	NR	NR	NR	NR	OR: 1.52 (0.96, 2.41)†	NR	..
Castro-Lima et al (2019)	NR	NR	NR	NR	26/39	15/37	OR: 2.89 (1.11–7.52)	OR: 2.59 (0.85–7.9)	Age, diabetes mellitus, SOFA score on the day of the first HAI and MDROS infection
Matos et al (2016) (Total)	14/20	20/34	NR	NR	NR	NR	OR: 1.63 (0.50–5.29)	NR	..
Matos et al (2016) (Pediatric)	1/4	11/20	NR	NR	NR	NR	OR: 0.28 (0.02–3.09)	NR	..
Matos et al (2016) (Adult)	13/16	10/14	NR	NR	NR	NR	OR: 1.73 (0.31–9.57)	NR	..
Costa et al (2015)‡	NR	NR	NR	NR	12/47	9/54	OR: 1.71 (0.65–4.52)†	NR	..
Islas-Muñoz et al (2018) (Total)	NR	NR	NR	NR	54/196	55/300	OR: 1.69 (1.07–2.65)	OR: 1.59 (0.99–2.65)	..
Karve et al (2018)	21/30	16/50	NR	NR	NR	NR	OR: 4.96 (1.86–13.23)	NR	..
Nassar et al (2021)	35/70	115/237	NR	NR	NR	NR	OR: 1.06 (0.62–1.81)†	NR	..
Prata-Rocha et al (2012)	NR	NR	NR	NR	21/49	8/24	OR: 1.50 (0.48–4.72)	NR	..
Seligman et al (2013) (Total)	28/59	34/81	NR	NR	NR	NR	OR: 1.25 (0.64–2.25)	NR	..
Carbapenem-resistant Gram-negative bacilli (CR-GNB)	..	..	..	..	..	..	..	..	..
Herrera et al (2021)***	61/308	34/463	NR	NR	77/308	44/463	HR: 3.9 (2.9–5.2)	HR: 1.89 (1.1–3.1)	***
Extremely drug-resistant organisms (XDRO)	..	..	..	..	..	..	..	..	..
Copaja-Corzo et al (2020)	25/40	16/84	NR	NR	NR	NR	OR: 7.08 (3.06–16.41)†	NR	..
Multidrug resistant Gram-negative bacilli (MDR-GNB)	..	..	..	..	..	..	..	..	..
Quillici et al (2021)	NR	NR	NR	NR	49/92	28/45	OR: 0.69 (0.33–1.43)	NR	..
Seligman et al (2013)	11/24	23/60	NR	NR	NR	NR	OR: 1.36 (0.52–3.54)†	NR	..

*CHF, congestive heart failure; HAI, hospital-acquired infection; HR, hazard ratio; NR, not reported; OR, odds ratio; R, resistant microorganism; RI, renal insufficiency; RR, risk ratio; S, susceptible microorganism; SOFA, sequential organ failure assessment.

Study	In-hospital lethality R (n/N)	In-hospital lethality S (n/N)	15-d lethality R (n/N)	15-d lethality S (n/N)	30-d lethality R (n/N)	30-d lethality S (n/N)	Unadjusted OR/RR/HR (95% CI)	Adjusted OR/RR/HR (95% CI)	Type of adjustment

†Comparative measure calculated with reported data.
‡Number of episodes reported (not patients).
§Isolates of MRSA and VRE were included.
¶More than two antimicrobials' agents, cardiopathy, presence of intravascular device, length of hospital stay before bacteraemia >7 d.
#675 out of 915 patients were included in the final analysis.
**Age, gender, congestive heart failure, renal insufficiency, chronic obstructive pulmonary disease, malignancy, AIDS, Foley catheter, mechanical ventilation, presence of intravascular device, hemodialysis, surgical procedure, use of corticosteroids, bacteraemia, inappropiate empirical treatment (SHOULD BE Bellísimo-Rodrigues et al (2006)
††21-d mortality.
‡‡28-d mortality.
§§Isolates of Carbapenem-resistant *Pseudomonas aeruginosa* and *Acinetobacter baumanni* were included.
¶¶Isolates of MDR *Pseudomonas aeruginosa* and *Acinetobacter baumannii* were included.(SHOULD BE QUILLICI)
##15 non-fermenting bacteria outside the scope (*Stenotrophomonas maltophilia* and non-specified *Pseudomonas* or *Acinetobacter*) were included in the final analysis.
***771 out of 1277 patients were included in the final analysis.
†††Relapse disease, refractory disease, respiratory source, nosocomial infection, intensive care unit admission, shock, PITT score ≥4, 7-d clinical response.

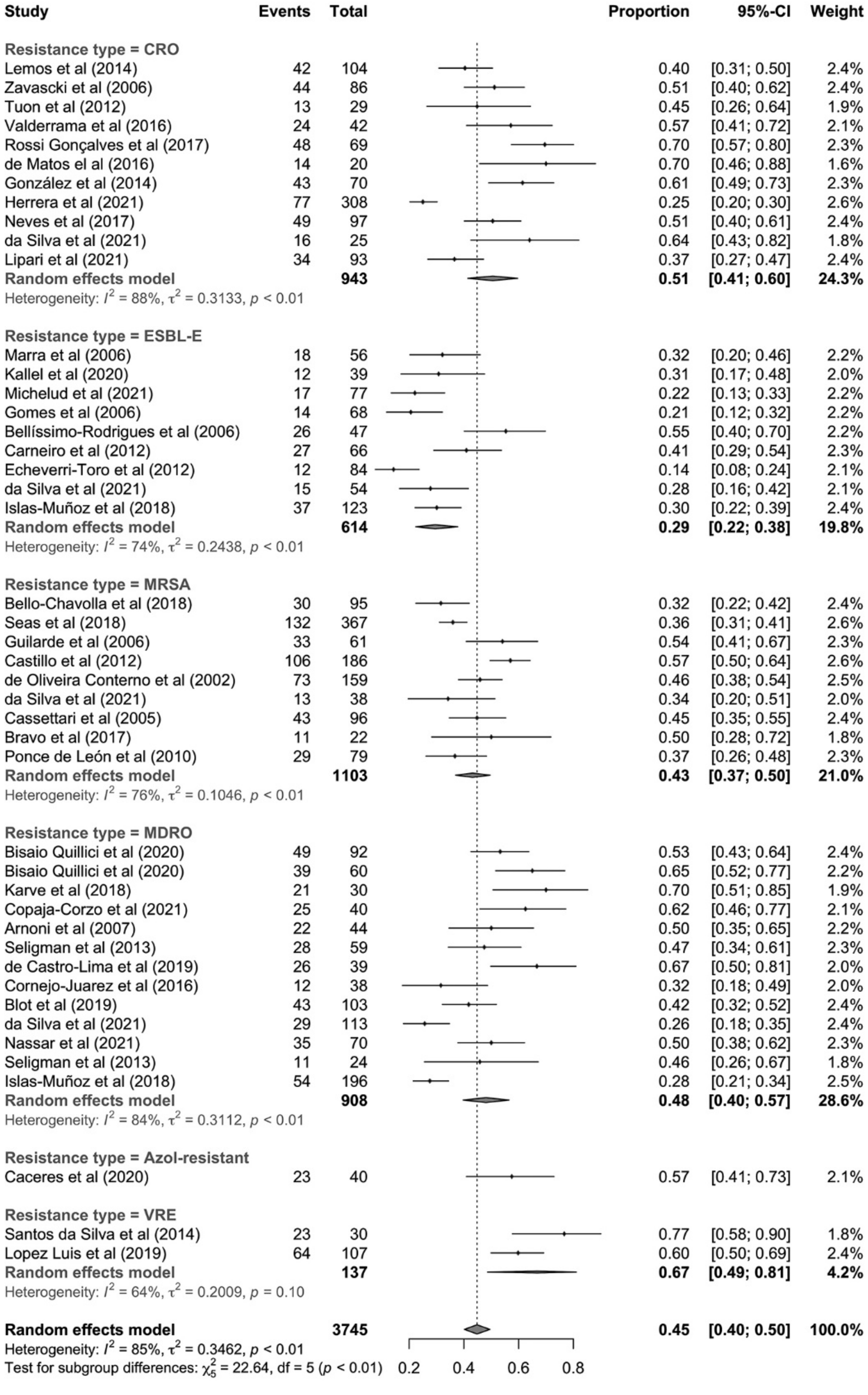

Appendix Figure 1. Crude lethality rate due to different type of antimicrobial resistance. Death-R: death in resistant group; Death-S: death in susceptible group; n: number of death. N: group size; OR: odds ratio; CI: confidence interval; CRO: Carbapenem resistant organisms; ESBL-E: extended spectrum β-lactamase producing Enterobacterales; MRSA: methicillin-resistant *Staphylococcus aureus*; MDRO: multidrug resistant organisms; VRE: Vancomycin-resistant *Enterococcus* spp.

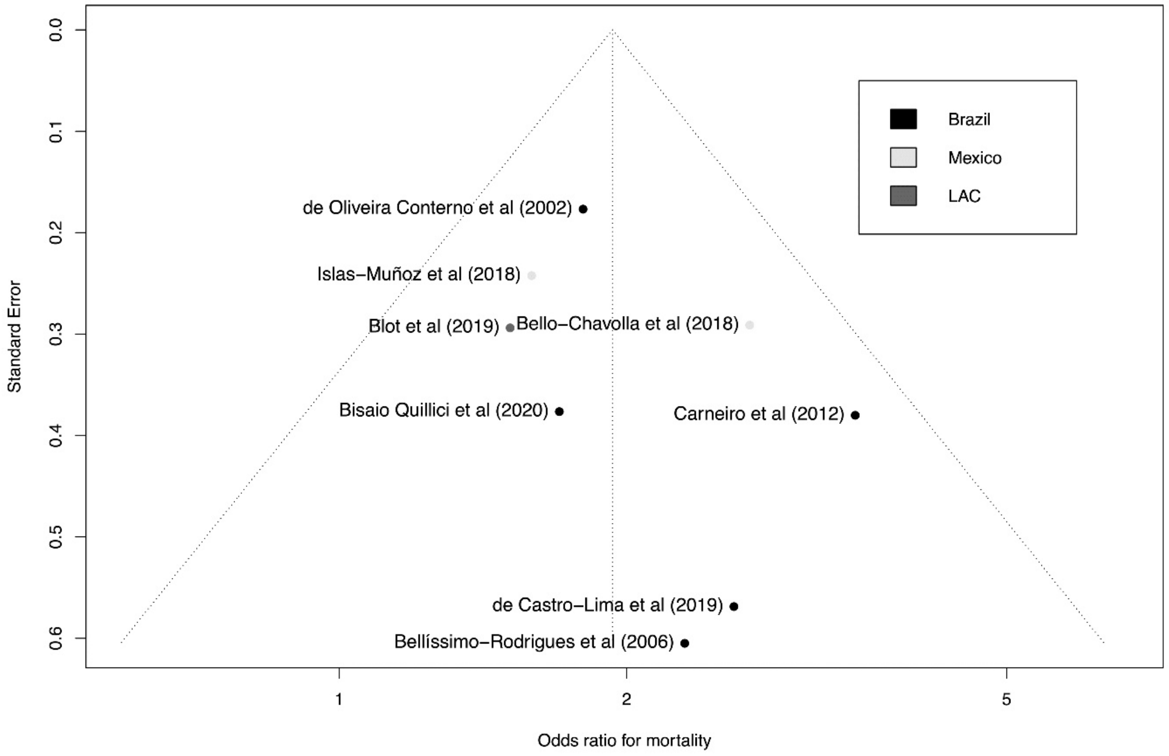

Appendix Figure 2. Funnel plot to assess bias in the studies with adjusted odds ratios for lethality between susceptible and resistant infections. LAC: Latin American and Carribean. Begg's test p-value: = 0.322.

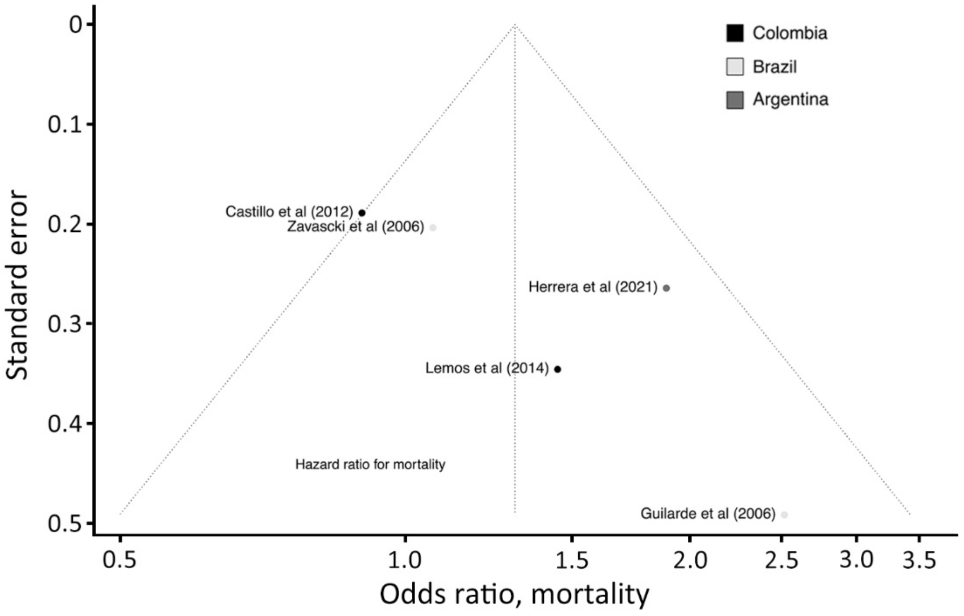

Appendix Figure 3. Funnel plot to assess bias in the studies with adjusted hazard ratios for lethality between susceptible and resistant infections. Begg's test p-value: = 0.141.

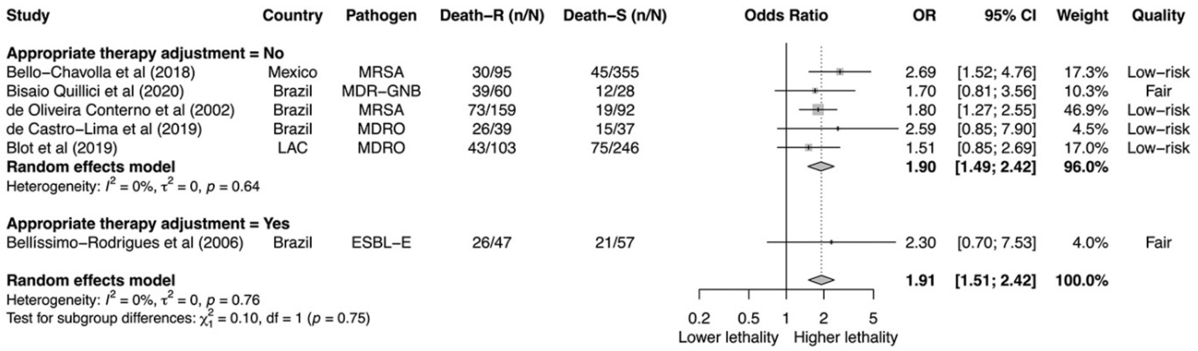

Appendix Figure 4. Association between resistance and mortality by studies that included appropriate empirical antibiotic treatment in the adjusted model. Adjusted odds ratios. We consider the definition of appropriate therapy given by each study author. Only studies that report adjusted odds ratios were included in this analysis. Death-R: death in the resistant group; Death-S: death in the susceptible group; n: number of deaths; N: group size; OR: odds ratio; CI: confidence interval; MRSA: methicillin-resistant Staphylococcus aureus; MDR-GNB: multidrug-resistant Gram-negative bacilli (including ESBL-E: Extended spectrum β-lactamase Enterobacterales, CRE: Carbapenem-resistant Enterobacterales, CRPA: Carbapenem-resistant *Pseudomonas aeruginosa,* CRAB: Carbapenem-resistant *Acinetobacter baumannii*); MDRO: multidrug-resistant organisms (including MRSA, VRE: Vancomycin-resistant *Enterococcus* spp, ESLB-E, CRE, CR-PA, CR-AB); LAC: Latin American and Caribbean countries.

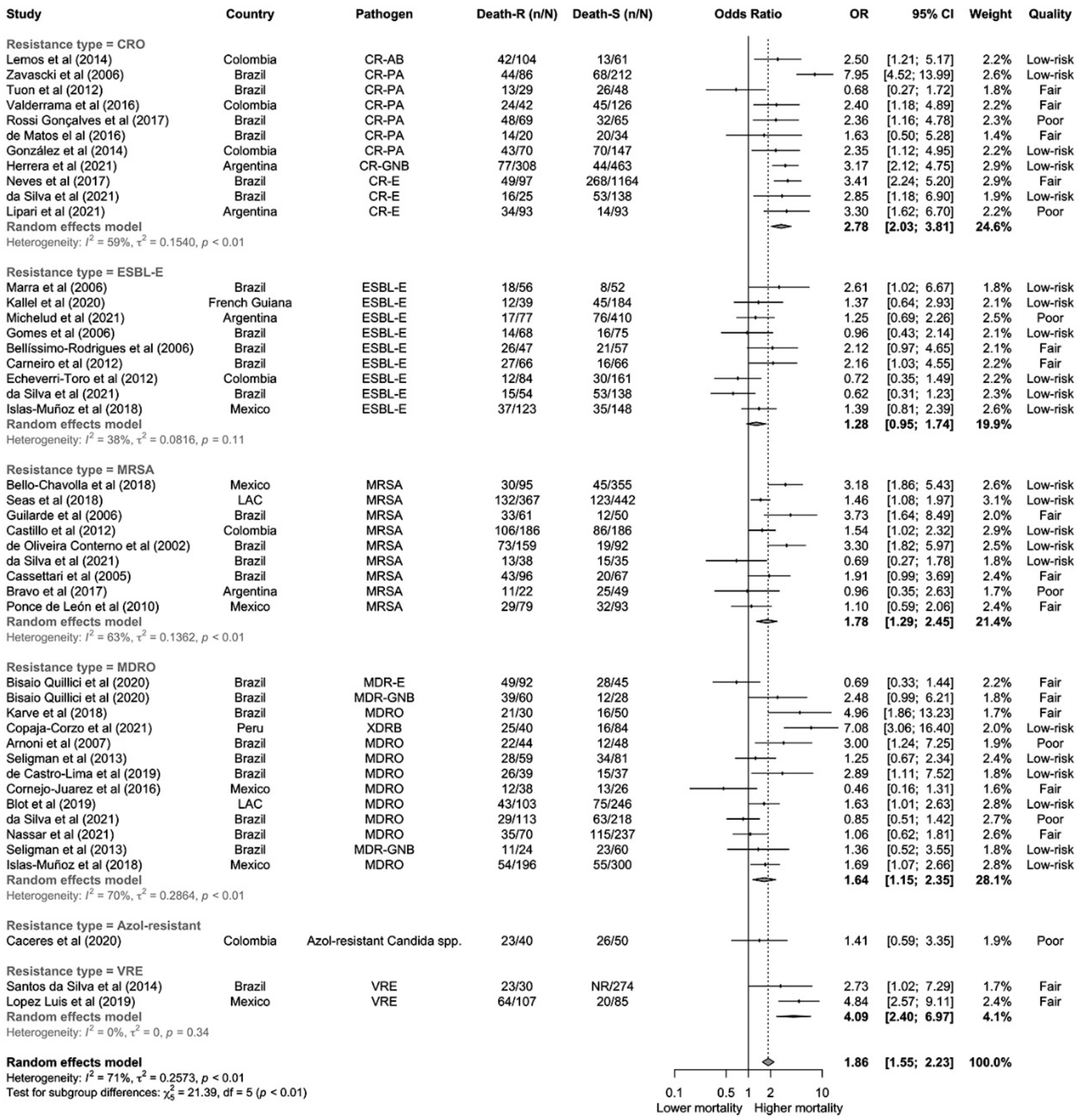

Appendix Figure 5. Forest plot summary of the unadjusted results (OR) for lethality by type of resistance. Death-R: death in resistant group; Death-S: death in susceptible group; n: number of death. N: group size; OR: odds ratio; CI: confidence interval; CR-AB: Carbapenem-resistant *Acinetobacter baumannii*; CR-PA: Carbapenem-resistant *Pseudomonas aeruginosa*; GNB-CR: Gram-negative bacilli carbapenem-resistant; CR-E: Carbapenem-resistant Enterobacterales; CRO: Carbapenen resistant organisms; ;ESBL-E: extended spectrum β-lactamase producing Enterobacterales; MRSA: methicillin-resistant *Staphylococcus aureus*; MDRO: multidrug resistant organisms; MDR-E: multidrug resistant Enterobacterales; MDR-GNB: multidrug resistant Gram-negative bacilli; XDRB: Extremely drug-resistant bacteria (XDRB); VRE: Vancomycin-resistant *Enterococcus* spp.; LAC: Latin American and Caribbean.

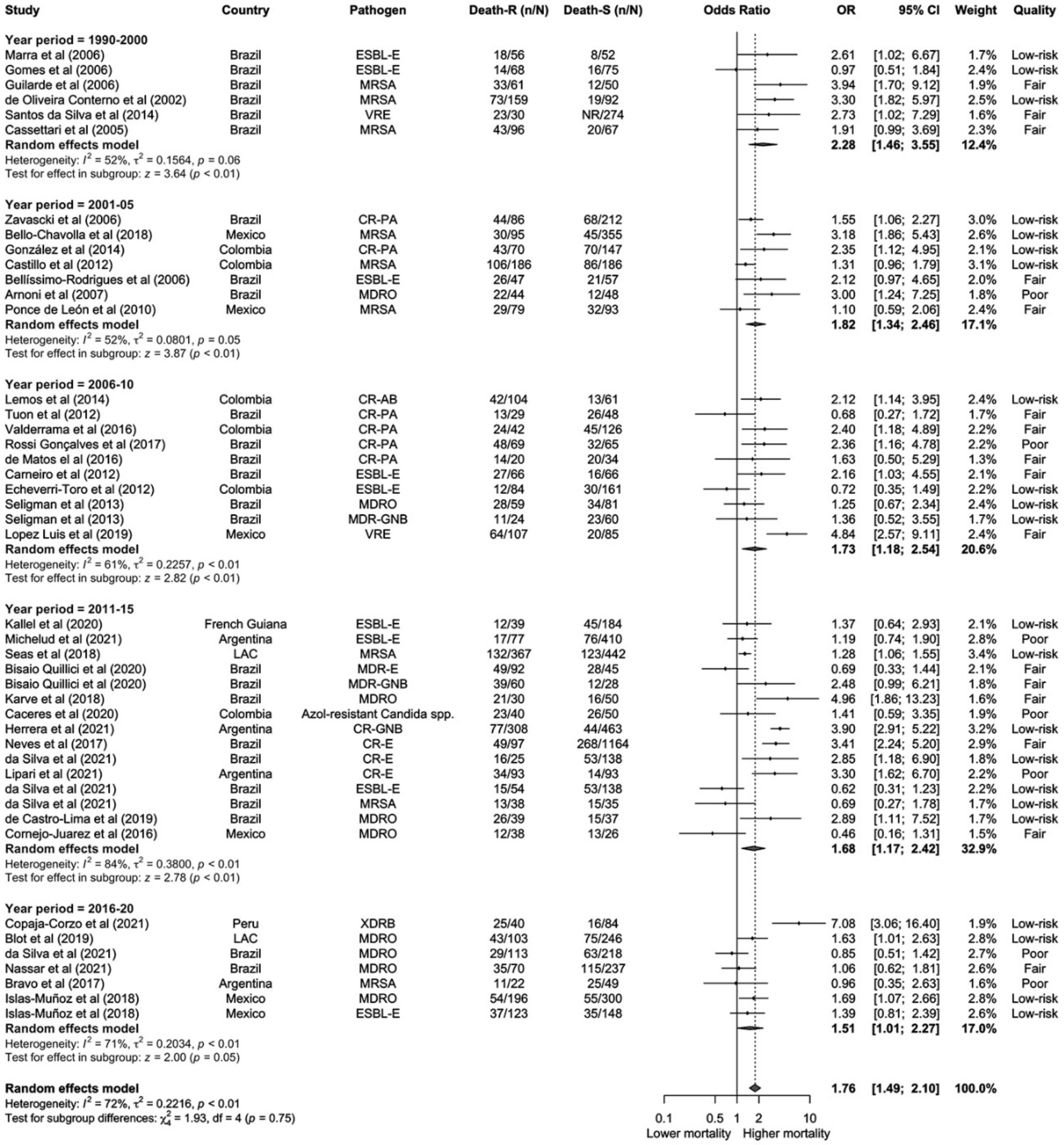

Appendix Figure 6. Forest plot summary of the unadjusted results (OR) for lethality by year period of study recruitment. Death-R: death in resistant group; Death-S: death in susceptible group; n: number of death. N: group size; OR: odds ratio; CI: confidence interval; ESBL-E: extended spectrum β-lactamase producing Enterobacterales; MRSA: methicillin-resistant *Staphylococcus aureus*; VRE: Vancomycin-resistant *Enterococcus* spp.; CR-PA: Carbapenem-resistant *Pseudomonas aeruginosa*; MDRO: multidrug resistant organisms; CR-AB: Carbapenem-resistant *Acinetobacter baumannii*; MDR-GNB: multidrug resistant Gram-negative bacilli; LAC: Latin American and Caribbean.

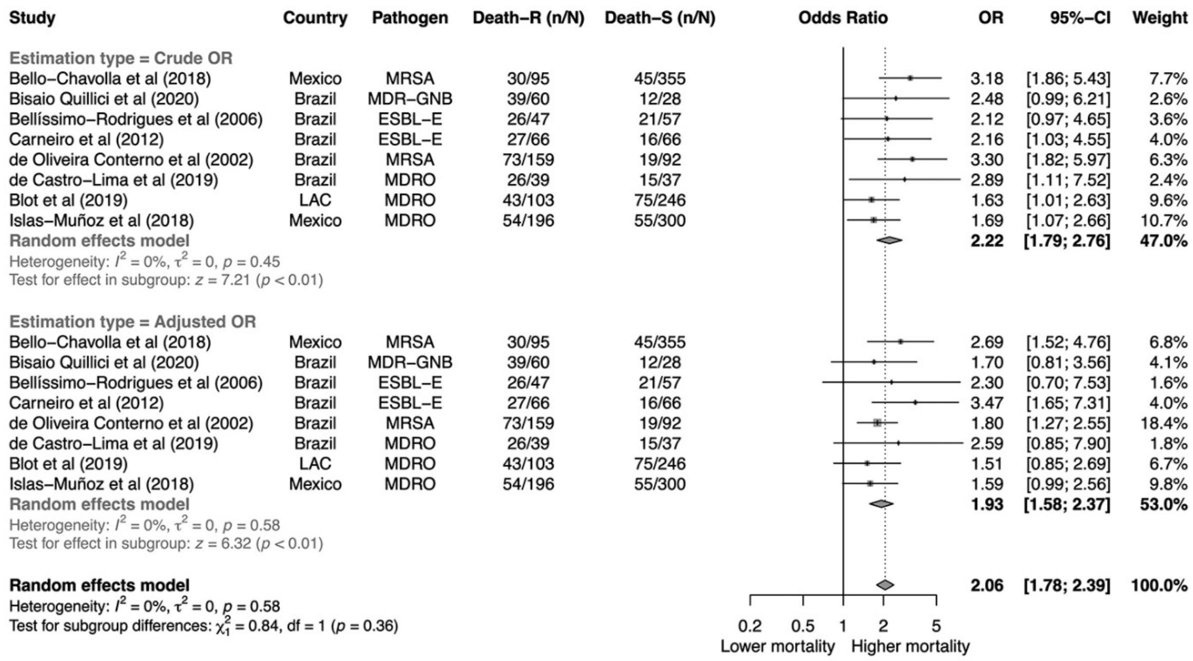

Appendix Figure 7. Subgroup analysis for lethality between crude and adjusted ORs. Studies reporting both adjusted and crude odds ratios were included in this analysis. Although no significant difference has been found between the subgroups (p = 0.360), the crude polled estimate tends to overestimate resistance-related lethality. Death-R: death in resistant group; Death-S: death in susceptible group; n: number of death. N: group size; OR: odds ratio; CI: confidence interval; MRSA: methicillin-resistant Staphylococcus aureus; MDR-GNB: multidrug resistant Gram-negative bacilli; ESBL-E: extended spectrum β-lactamase enterobacteriaceae spp.; MDRO: multidrug resistant organism.

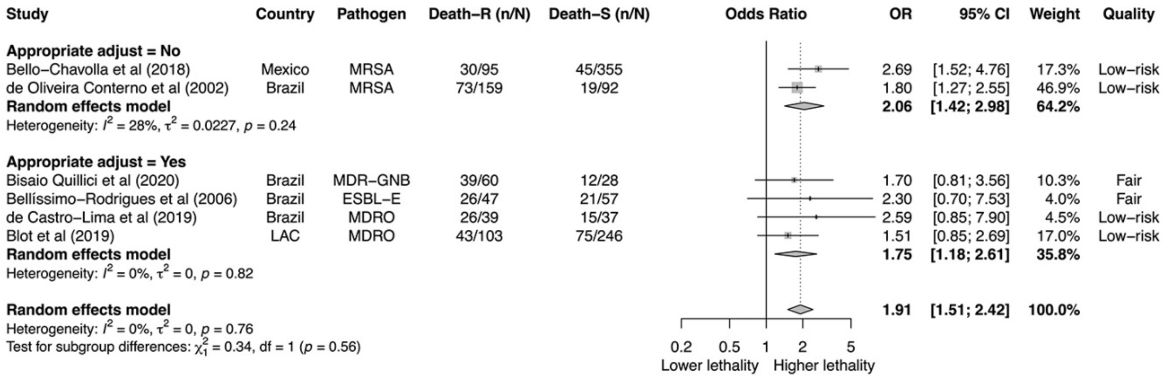

Appendix Figure 8. Association between resistance and lethality by appropriate adjustment. OR: odds ratio; CI: confidence interval; MRSA: methicillin-resistant *Staphylococcus aureus*; MDR-GNB: multidrug resistant Gram-negative bacilli; ESBL-E: extended spectrum β-lactamase producing Enterobacterales; MDRO: multidrug resistant organism. We consider appropriate adjustment between lethality and resistance when the study considers in the final model at least one variable in each category: a) variables related to the patients' baseline status, b) variables related to the infection; and c) variables related to the treatment (*68*).

References

1. Barrero LI, Castillo JS, Leal AL, Sánchez R, Cortés JA, Álvarez CA, et al. Impacto económico de la resistencia a meticilina en pacientes con bacteriemia por *Staphylococcus aureus* en hospitales de Bogotá. Biomedica. 2014;34. 10.7705/biomedica.v34i3.1692 https://doi.org/10.7705/biomedica.v34i3.1692

2. Cartaxo Salgado FX, Carneiro Gonçalves J, Monteiro De Souza C, Barbosa Da Silva N, Gavilanes Sánchez TE, Gomes de Oliveira Karnikowski M. Cost of antimicrobial treatment of patients infected with multidrug-resistant organisms in the Intensive Care Unit. Medicina (B Aires). 2011;71:531–5. PubMed

3. Lopez Luis B, Lambraño-Castillo D, Ortiz-Brizuela E, Ramirez-Fontes A, Estrella Tovar-Calderon Y, Javier Leal-Vega F, et al. 573. Enterococcal Bacteremia in a Tertiary Care Center in Mexico: A Retrospective Analysis Focus on Vancomycin-Resistant *E. faecium* and Ampicillin-Resistant *E. faecalis*. Open Forum Infect Dis. 2019;6(Suppl 2):S270–1. https://doi.org/10.1093/ofid/ofz360.642

4. Lucena A, Dalla Costa LM, Nogueira KS, Matos AP, Gales AC, Paganini MC, et al. Nosocomial infections with metallo-beta-lactamase-producing *Pseudomonas aeruginosa*: molecular epidemiology, risk factors, clinical features and outcomes. J Hosp Infect. 2014;87:234–40. PubMed https://doi.org/10.1016/j.jhin.2014.05.007

5. Fiorentin Vandresen D, Carolina Lucio L, Shigueyasu Yamada R, Paula Vieira A, Ani Caovilla Follador F, Pitt Benedetti V, et al. Associated factors of *Acinetobacter baumannii* complex in hospitalized patients: A case-control study. J Infect Dev Ctries. 2021;15:73–80. PubMed https://doi.org/10.3855/jidc.13525

6. Furtado GHC, Bergamasco MD, Menezes FG, Marques D, Silva A, Perdiz LB, et al. Imipenem-resistant *Pseudomonas aeruginosa* infection at a medical-surgical intensive care unit: risk factors and mortality. J Crit Care. 2009;24:625.e9–14. PubMed https://doi.org/10.1016/j.jcrc.2009.03.006

7. Mano A, Clemente W. Bacteremias por serratia marcescens resistentes a carbapenêmicos: ameaça maior que bacteremias por outras enterobactérias resistentes a carbapenêmicos? Presented at: XVII Congresso Brasileiro de Controles de Infeccao e Epidemiología Hospitalar. 2021 May 13–15; São Paulo, Brazil.

8. Pinheiro MRS, Lacerda HR, Melo RGL, Maciel MA. *Pseudomonas aeruginosa* infections: factors relating to mortality with emphasis on resistance pattern and antimicrobial treatment. Braz J Infect Dis. 2008;12:509–15. PubMed https://doi.org/10.1590/S1413-86702008000600013

9. Michelud C, Salmeron Olsina A, Gordóvil M, De Wouters L, Vallejo M. Mortalidad de las bacteriemias por escherichia coli en pacientes mayores de 65 años en un hospital privado. revisión años 2014–2018. Presented at: 21 Congreso de La Sociedad Argentina de Infectología (SADI). 2021 Oct 25–27; Buenos Aires, Argentina.

10. Dantas RC, Ferreira ML, Gontijo-Filho PP, Ribas RM. *Pseudomonas aeruginosa* bacteraemia: independent risk factors for mortality and impact of resistance on outcome. J Med Microbiol. 2014;63:1679–87. PubMed https://doi.org/10.1099/jmm.0.073262-0

11. Freire MP, Pierrotti LC, Filho HHC, Ibrahim KY, Magri AS, Bonazzi PR, et al. Infection with *Klebsiella pneumoniae* carbapenemase (KPC)-producing *Klebsiella pneumoniae* in cancer patients. Eur J Clin Microbiol Infect Dis. 2015;34:277–86. PubMed https://doi.org/10.1007/s10096-014-2233-5

12. Bento Talizin T, Dantas de Maio Carrilho CM, Magalhães Carvalho Grion C, Tibery Queiroz Cardoso L, Toshiyuki Tanita M, Boll KM, et al. Polymyxin for treatment of ventilator-associated pneumonia in a setting of high carbapenem resistance. PLoS One. 2020;15:e0237880. PubMed https://doi.org/10.1371/journal.pone.0237880

13. Cassettari V, Silveira IR. IIda LIS, Fernandes JB, Santos RB dos. Comparação da mortalidade em pacientes colonizados e infectados por *Acinetobacter baumannii* em uti clínico-cirúrgica de adultos. Journal of Infection Control. 2012;1:s107.

14. Cezário RC, Duarte De Morais L, Ferreira JC, Costa-Pinto RM, da Costa Darini AL, Gontijo-Filho PP. Nosocomial outbreak by imipenem-resistant metallo-beta-lactamase-producing *Pseudomonas aeruginosa* in an adult intensive care unit in a Brazilian teaching hospital. Enferm Infecc Microbiol Clin. 2009;27:269–74. PubMed https://doi.org/10.1016/j.eimc.2008.09.009

15. Cusmano L, Valdivia Dahl A, Viteri M, Terusi A, Mattiello Sacchi V, Shimank E, et al. La infeccion por klebsiella productora de carbapenemasas en una unidad de cuidados intensivos de adultos mayores: ¿como impacta en los indices de calidad de atencion? Presented at: 16 Congreso Panamericano de Infectolgía; 30 Congreso Chileno de Infectología. 2013 May 28–Jun 1; Santiago, Chile.

16. Pinoni M. Bacteriemias en receptores de organo solido: incidencia, etiologia, factores de riesgo y evolucion. Estudio multicentrico. Presented at: 21 Congreso de La Sociedad Argentina de Infectología (SADI). 2021 Oct 25–27; Buenos Aires, Argentina.

17. Superti SV, Augusti G, Zavascki AP. Risk factors for and mortality of extended-spectrum-beta-lactamase-producing *Klebsiella pneumoniae* and *Escherichia coli* nosocomial bloodstream

infections. Rev Inst Med Trop São Paulo. 2009;51:211–6. PubMed https://doi.org/10.1590/S0036-46652009000400006

18. Romi RP, Freire M, Bittencourt DP, Bonazzi PR, Hatanaka VMA, Ibrahim KY, et al. Does the Gram-negative Bacteria Colonization Impact on the Risk of Develop Infection by Those Organisms During Neutropenia? Open Forum Infect Dis. 2017;4(Suppl 1):S705–6. https://doi.org/10.1093/ofid/ofx163.1893

19. da Silva KE, Baker S, Croda J, Nguyen TNT, Boinett CJ, Barbosa LS, et al. Risk factors for polymyxin-resistant carbapenemase-producing Enterobacteriaceae in critically ill patients: An epidemiological and clinical study. Int J Antimicrob Agents. 2020;55:105882. PubMed https://doi.org/10.1016/j.ijantimicag.2020.105882

20. Arnoni MV, Berezin EN, Martino MDV. Risk factors for nosocomial bloodstream infection caused by multidrug resistant gram-negative bacilli in pediatrics. Braz J Infect Dis. 2007;11:267–71. PubMed https://doi.org/10.1590/S1413-86702007000200020

21. Bellíssimo-Rodrigues F, Gomes ACF, Passos ADC, Achcar JA, Perdoná GS, Martinez R. Clinical outcome and risk factors related to extended-spectrum beta-lactamase-producing *Klebsiella* spp. infection among hospitalized patients. Mem Inst Oswaldo Cruz. 2006;101:415–21. PubMed https://doi.org/10.1590/S0074-02762006000400012

22. Bello-Chavolla OY, Bahena-Lopez JP, Garciadiego-Fosass P, Volkow P, Garcia-Horton A, Velazquez-Acosta C, et al. Bloodstream infection caused by *S. aureus* in patients with cancer: a 10-year longitudinal single-center study. Support Care Cancer. 2018;26:4057–65. PubMed https://doi.org/10.1007/s00520-018-4275-1

23. Quillici MCB, Resende DS, Gonçalves IR, Royer S, Sabino SS, Almeida VF, et al. Gram-negative bacilli bacteremia: a 7 year retrospective study in a referral Brazilian tertiary-care teaching hospital. J Med Microbiol. 2021;70. 10.1099/jmm.0.001277 PubMed https://doi.org/10.1099/jmm.0.001277

24. Blot S, Antonelli M, Arvaniti K, Blot K, Creagh-Brown B, de Lange D, et al.; Abdominal Sepsis Study (AbSeS) group on behalf of the Trials Group of the European Society of Intensive Care Medicine. Epidemiology of intra-abdominal infection and sepsis in critically ill patients: “AbSeS”, a multinational observational cohort study and ESICM Trials Group Project. Intensive Care Med. 2019;45:1703–17. PubMed https://doi.org/10.1007/s00134-019-05819-3

25. Bravo M, Altamirano N, Alvarez S, Maresca J, Adra M, Carrion N, et al. Características clínicas y epidemiológicas de los pacientes con bacteriemias por stafilococcus aureus en un hospital general. Presented at: 17 Congreso de La Sociedad Argentina de Infectilogía (SADI). 2017 Jun 15–17; Buenos Aires, Argentina.

26. Caceres DH, Rivera SM, Armstrong PA, Escandon P, Chow NA, Ovalle MV, et al. Case-case comparison of *Candida auris* versus other *Candida* species bloodstream infections: Results of an outbreak investigation in Colombia. Mycopathologia. 2020;185:917–23. PubMed https://doi.org/10.1007/s11046-020-00478-1

27. Carneiro IC do RS. Silva DL da. Epidemiologia das infecções de corrente sanguínea por enterobactérias produtoras esbl: estudo caso-controle em unidade neonatal do brasil. Journal of Infection Control. 2012;1:S36.

28. Cassettari VC, Strabelli T, Medeiros EAS. *Staphylococcus aureus* bacteremia: what is the impact of oxacillin resistance on mortality? Braz J Infect Dis. 2005;9:70–6. PubMed https://doi.org/10.1590/S1413-86702005000100012

29. Castillo JS, Leal AL, Cortes JA, Alvarez CA, Sanchez R, Buitrago G, et al.; GREBO. Mortality among critically ill patients with methicillin-resistant *Staphylococcus aureus* bacteremia: a multicenter cohort study in Colombia. Rev Panam Salud Publica. 2012;32:343–50. PubMed https://doi.org/10.1590/S1020-49892012001100004

30. Copaja-Corzo C, Hueda-Zavaleta M, Benites-Zapata VA, Rodriguez-Morales AJ. Antibiotic use and fatal outcomes among critically ill patients with COVID-19 in Tacna, Peru. Antibiotics (Basel). 2021;10:959. PubMed https://doi.org/10.3390/antibiotics10080959

31. Cornejo-Juárez P, Vilar-Compte D, García-Horton A, López-Velázquez M, Ñamendys-Silva S, Volkow-Fernández P. Hospital-acquired infections at an oncological intensive care cancer unit: differences between solid and hematological cancer patients. BMC Infect Dis. 2016;16:274. PubMed https://doi.org/10.1186/s12879-016-1592-1

32. Cornejo-Juárez P, Vilar-Compte D, Pérez-Jiménez C, Ñamendys-Silva SA, Sandoval-Hernández S, Volkow-Fernández P. The impact of hospital-acquired infections with multidrug-resistant bacteria in an oncology intensive care unit. Int J Infect Dis. 2015;31:31–4. PubMed https://doi.org/10.1016/j.ijid.2014.12.022

33. Correa L, Martino MDV, Siqueira I, Pasternak J, Gales AC, Silva CV, et al. A hospital-based matched case-control study to identify clinical outcome and risk factors associated with carbapenem-resistant *Klebsiella pneumoniae* infection. BMC Infect Dis. 2013;13:80. PubMed https://doi.org/10.1186/1471-2334-13-80

34. da Silva NCZ, da Rocha JA, do Valle FM, Silva ASDN, Ehrlich S, Martins IS. The impact of ageing on the incidence and mortality rate of bloodstream infection: A hospital-based case-cohort study in a tertiary public hospital of Brazil. Trop Med Int Health. 2021;26:1276–84. PubMed https://doi.org/10.1111/tmi.13650

35. Oliveira da Silva AR, Nagem LP, Oliveira C, Sardinha G, Salgado DR, Lima E C. Fatores de riscos associados a infecção por patógenos mdr em pacientes internados em terapia. Presented at: XVII Congresso Brasileiro de Controles de Infeccao e Epidemiología Hospitalar. 2021 May 13–15; São Paulo, Brazil.

36. Castro-Lima VAC, Borges IC, Joelsons D, Sales VVT, Guimaraes T, Ho YL, et al. Impact of human immunodeficiency virus infection on mortality of patients who acquired healthcare associated-infection in critical care unit. Medicine (Baltimore). 2019;98:e15801. PubMed https://doi.org/10.1097/MD.0000000000015801

37. Matos EC, Matos HJ, Conceição ML, Rodrigues YC, Carneiro IC, Lima KV. Clinical and microbiological features of infections caused by *Pseudomonas aeruginosa* in patients hospitalized in intensive care units. Rev Soc Bras Med Trop. 2016;49:305–11. PubMed https://doi.org/10.1590/0037-8682-0446-2015

38. Costa PO, Atta EH, Silva AR. Infection with multidrug-resistant gram-negative bacteria in a pediatric oncology intensive care unit: risk factors and outcomes. J Pediatr (Rio J). 2015;91:435–41. PubMed https://doi.org/10.1016/j.jped.2014.11.009

39. De Vedia L, Lista N, Di Virgilio E, Rodriguez A, Piovano G, Cisneros JC, et al. Infecciones graves de la comunidad por *Staphylococcus aureus* en terapia intensiva: experiencia en los últimos 15 años. Presented at: 17 Congreso de La Sociedad Argentina de Infectilogía (SADI). 2017 Jun 15–17; Buenos Aires, Argentina.

40. Ducatenzeiler L, Benso J, Moltrasio M, Greco G, Barcan L, Staneloni I. Resistencia a colistín: aumento de incidencia y pocas alternativas terapéuticas. Presented at: 17 Congreso de La Sociedad Argentina de Infectilogía (SADI). 2017 Jun 15–17; Buenos Aires, Argentina.

41. Echeverri-Toro LM, Rueda ZV, Maya W, Agudelo Y, Ospina S. [Multidrug-resistant *Klebsiella pneumoniae*, predisposing factors and associated mortality in a tertiary-care hospital in Colombia]. Rev Chilena Infectol. 2012;29:175–82. PubMed https://doi.org/10.4067/S0716-10182012000200009

42. Gañete M, Playonero G, Espinosa L, J Sablich, V Vergara, J Crespo, et al. Análisis descriptivo de bacteriemias por staphylococcus aureus en adultos asistidos en un hospital general de agudos. Presented at: 21 Congreso de La Sociedad Argentina de Infectología (SADI). 2021 Oct 25–27; Buenos Aires, Argentina.

43. Gentile Á, Bakir J, Ensinck G, Cancellara A, Casanueva EV, Firpo V, et al.; Grupo de Trabajo de Staphylococcus aureus. Community-acquired methicillin-resistant *Staphylococcus aureus* infections: hospitalization and case fatality risk in 10 pediatric facilities in Argentina. Arch Argent Pediatr. 2018;116:e47–53. PubMed

44. Gomes CC, Vormittag E, Santos CR, Levin AS. Nosocomial infection with cephalosporin-resistant *Klebsiella pneumoniae* is not associated with increased mortality. Infect Control Hosp Epidemiol. 2006;27:907–12. PubMed https://doi.org/10.1086/507276

45. González AL, Leal AL, Cortés JA, Sánchez R, Barrero LI, Castillo JS, et al. [Effect of adequate initial antimicrobial therapy on mortality in critical patients with *Pseudomonas aeruginosa* bacteremia]. Biomedica. 2014;34(Suppl 1):58–66. PubMed

46. Guilarde AO, Turchi MD, Martelli CMT, Primo MGB. *Staphylococcus aureus* bacteraemia: incidence, risk factors and predictors for death in a Brazilian teaching hospital. J Hosp Infect. 2006;63:330–6. PubMed https://doi.org/10.1016/j.jhin.2006.02.011

47. Herrera F, Laborde A, Jordán R, Berruezo L, Roccia Rossi I, Valledor A, et al. Current Epidemiology of Bacteremia in Patients with Hematological Malignancies and Hematopoietic Stem Cell Transplantation and the Impact of Antibiotic Resistance on Survival. Presented at: 31st European Congress of Clinical Microbiology and Infectious Diseases. 2021 Jul 9–12 [online].

48. Islas-Muñoz B, Volkow-Fernández P, Ibanes-Gutiérrez C, Villamar-Ramírez A, Vilar-Compte D, Cornejo-Juárez P. Bloodstream infections in cancer patients. Risk factors associated with mortality. Int J Infect Dis. 2018;71:59–64. PubMed https://doi.org/10.1016/j.ijid.2018.03.022

49. Kallel H, Houcke S, Resiere D, Roy M, Mayence C, Mathien C, et al. Epidemiology and prognosis of intensive care unit-acquired bloodstream infection. Am J Trop Med Hyg. 2020;103:508–14. PubMed https://doi.org/10.4269/ajtmh.19-0877

50. Karve S, Ryan K, Peeters P, Baelen E, Rojas-Farreras S, Potter D, et al. The impact of initial antibiotic treatment failure: Real-world insights in patients with complicated urinary tract infection. J Infect. 2018;76:121–31. PubMed https://doi.org/10.1016/j.jinf.2017.11.001

51. Lemos EV, de la Hoz FP, Alvis N, Einarson TR, Quevedo E, Castañeda C, et al. Impact of carbapenem resistance on clinical and economic outcomes among patients with *Acinetobacter baumannii* infection in Colombia. Clin Microbiol Infect. 2014;20:174–80. PubMed https://doi.org/10.1111/1469-0691.12251

52. Lipari F, Hernández D, Cometto A, Ruiz S, Irrazabal G, Caeiro JP, et al. Caracterización clínica y microbiológica de bacteriemias por enterobacterales productoras de carbapenemasas. eficacia de ceftazidima-avibactam como opción terapéutica. Presented at: 21 Congreso de La Sociedad Argentina de Infectología (SADI). 2021 Oct 25–27; Buenos Aires, Argentina.

53. Marra AR, Wey SB, Castelo A, Gales AC, Cal RG, Filho JR, et al. Nosocomial bloodstream infections caused by *Klebsiella pneumoniae*: impact of extended-spectrum beta-lactamase

(ESBL) production on clinical outcome in a hospital with high ESBL prevalence. BMC Infect Dis. 2006;6:24. PubMed https://doi.org/10.1186/1471-2334-6-24

54. Moreira MR, Cardoso RL, Almeida AB, Gontijo Filho PP. Risk factors and evolution of ventilator-associated pneumonia by *Staphylococcus aureus* sensitive or resistant to oxacillin in patients at the intensive care unit of a Brazilian university hospital. Braz J Infect Dis. 2008;12:499–503. PubMed https://doi.org/10.1590/S1413-86702008000600011

55. Nassar AP, Bezerra IL, Rodrigues M, De Sousa EC, De Lucas PH, De Carvalho A, et al. Impact of intensive care unit acquired infections caused by multidrug resistant organisms on costs: Preliminary findings of a Brazilian multicenter cohort study. Presented at: 40th International Symposium on Intensive Care and Emergency Medicine. 2021 Aug 31–Sep 3; Brussels, Belgium.

56. Naves KSC, Vaz da Trindade N, Gontijo Filho PP. Methicillin-resistant *Staphylococcus aureus* bloodstream infection: risk factors and clinical outcome in non-intensive-care units. Rev Soc Bras Med Trop. 2012;45:189–93. PubMed https://doi.org/10.1590/S0037-86822012000200010

57. Neves LE, dos Santos LJ. Análise do risco de infecção por enterobactérias resistentes a carbapenêmicos em hospital de grande porte referência em urgência e emergência de belo horizonte. Presented at: Competências Em Controle de Infecção Hospitalar (CCIH) 2017; MBA Gestão Em Saúde e Controle de Infecção Hospitalar: Jacareí Instituto Nacional de Ensino Superior e Pesquisa. 2017; Rio de Janeiro, Brazil.

58. Paternina-de la Ossa R, Prado SID, Cervi MC, Lima DAFDS, Martinez R, Bellissimo-Rodrigues F. Is community-associated methicillin-resistant *Staphylococcus aureus* (CA-MRSA) an emerging pathogen among children in Brazil? Braz J Infect Dis. 2018;22:371–6. PubMed https://doi.org/10.1016/j.bjid.2018.10.276

59. Ponce-de-León A, Camacho-Ortiz A, Macías AE, Landín-Larios C, Villanueva-Walbey C, Trinidad-Guerrero D, et al. Epidemiology and clinical characteristics of *Staphylococcus aureus* bloodstream infections in a tertiary-care center in Mexico City: 2003-2007. Rev Invest Clin. 2010;62:553–9. PubMed

60. Porto JP, Santos RO, Gontijo Filho PP, Ribas RM. Active surveillance to determine the impact of methicillin resistance on mortality in patients with bacteremia and influences of the use of antibiotics on the development of MRSA infection. Rev Soc Bras Med Trop. 2013;46:713–8. PubMed https://doi.org/10.1590/0037-8682-0199-2013

61. Prata-Rocha ML, Gontijo-Filho PP, Melo GB. Factors influencing survival in patients with multidrug-resistant *Acinetobacter baumannii* infection. Braz J Infect Dis. 2012;16:237–41. PubMed

62. Rossi Gonçalves I, Dantas RCC, Ferreira ML, Batistão DWDF, Gontijo-Filho PP, Ribas RM. Carbapenem-resistant *Pseudomonas aeruginosa*: association with virulence genes and biofilm formation. Braz J Microbiol. 2017;48:211–7. PubMed https://doi.org/10.1016/j.bjm.2016.11.004

63. da Silva NS, Muniz VD, Estofolete CF, Furtado GHC, Rubio FG. Identification of temporal clusters and risk factors of bacteremia by nosocomial vancomycin-resistant enterococci. Am J Infect Control. 2014;42:389–92. PubMed https://doi.org/10.1016/j.ajic.2013.11.010

64. Seas C, Garcia C, Salles MJ, Labarca J, Luna C, Alvarez-Moreno C, et al.; Latin America Working Group on Bacterial Resistance. *Staphylococcus aureus* bloodstream infections in Latin America: results of a multinational prospective cohort study. J Antimicrob Chemother. 2018;73:212–22. PubMed https://doi.org/10.1093/jac/dkx350

65. Seligman R, Ramos-Lima LF, Oliveira VA, Sanvicente C, Sartori J, Pacheco EF. Risk factors for infection with multidrug-resistant bacteria in non-ventilated patients with hospital-acquired pneumonia. J Bras Pneumol. 2013;39:339–48. PubMed https://doi.org/10.1590/S1806-37132013000300011

66. Tuon FF, Gortz LW, Rocha JL. Risk factors for pan-resistant *Pseudomonas aeruginosa* bacteremia and the adequacy of antibiotic therapy. Braz J Infect Dis. 2012;16:351–6. PubMed https://doi.org/10.1016/j.bjid.2012.06.009

67. Valderrama SL, González PF, Caro MA, Ardila N, Ariza B, Gil F, et al. Factores de riesgo para bacteriemia adquirida en el hospital por *Pseudomonas aeruginosa* resistente a carbapenémicos en un hospital colombiano. Biomedica. 2016;36. 10.7705/biomedica.v36i2.2784 PubMed https://doi.org/10.7705/biomedica.v36i2.2784

68. Zavascki AP, Barth AL, Gonçalves ALS, Moro AL, Fernandes JF, Martins AF, et al. The influence of metallo-β-lactamase production on mortality in nosocomial *Pseudomonas aeruginosa* infections. J Antimicrob Chemother. 2006;58:387–92. PubMed https://doi.org/10.1093/jac/dkl239

69. Araya S, Galeano F, Amarilla S, González N, Apodaca S, Lovera D, et al. Prognostic factors of severity of invasive community acquired *Staphylococcus aureus* infections in children. Arch Argent Pediatr. 2019;117:381–7. PubMed

70. de Oliveira Conterno L, Wey SB, Castelo A. *Staphylococcus aureus* bacteremia: comparison of two periods and a predictive model of mortality. Braz J Infect Dis. 2002;6:288–97. PubMed

71. Pinhati HMS, Casulari LA, Souza ACR, Siqueira RA, Damasceno CMG, Colombo AL. Outbreak of candidemia caused by fluconazole resistant *Candida parapsilosis* strains in an intensive care unit. BMC Infect Dis. 2016;16:433. PubMed https://doi.org/10.1186/s12879-016-1767-9

72. Zhang J, Yu KF. What's the relative risk? A method of correcting the odds ratio in cohort studies of common outcomes. JAMA. 1998;280:1690–1. PubMed https://doi.org/10.1001/jama.280.19.1690

73. Shor E, Roelfs D, Vang ZM. The "Hispanic mortality paradox" revisited: Meta-analysis and meta-regression of life-course differentials in Latin American and Caribbean immigrants' mortality. Soc Sci Med. 2017;186:20–33. PubMed https://doi.org/10.1016/j.socscimed.2017.05.049
